# The role of complement in the clinical course of hepatocellular carcinoma

**DOI:** 10.1002/iid3.569

**Published:** 2021-11-23

**Authors:** Xinye Qian, Zhoujing Yang, Lu Gao, Yipiao Liu, Jun Yan

**Affiliations:** ^1^ Center of Hepatobiliary Pancreatic Disease, Beijing Tsinghua Changgung Hospital Tsinghua University Beijing China; ^2^ Department of Anesthesiology, Huashan Hospital Fudan University Shanghai China

**Keywords:** complement, hepatocellular carcinoma, immune infiltration, Kaplan–Meier plotter, prognosis

## Abstract

**Background:**

The complement system, an innate immune system, may either play an antitumor role, or promote tumorigenesis and cancer progression in different kinds of cancer. The function of complement in hepatocellular carcinoma (HCC) is unclear.

**Methods:**

The gene expressions of the complement system were based on data obtained from TCGA and GEO. We explored gene expressions, mutation, enrichment analysis, clinicopathology, patients' outcome, and immune infiltration via Gepia2, cBioPortal, Metascape, UALCAN, Kaplan–Meier Plotter, and TIMER 2.

**Results:**

Five complement genes, including C1R, C6, C7, CFP, and CFHR3, were not only found to be significantly downregulated in HCC samples compared with normal liver samples, but also found to be significantly associated with overall survival, disease‐free survival, and progress‐free survival in HCC patients. In addition, lower mRNA expression of C1R, C6, C7, and CFHR3 were found correlated with advanced cancer stages and higher tumor grades in HCC patients. Also, the expression levels of CFP were correlated with many sets of immune markers of tumor immune cells, such as those of CD8+ T cells, CD4+ T cells, B cells, M2 macrophages, neutrophils, DCs, Th1 cells, Th2 cells, and T cell exhaustion in HCC. Based on that, we developed a prognostic model for HCC patients—Riskscore = (−0.0053)*C6+(−0.0498)*C7+(−0.1045)*CFHR3.

**Conclusion:**

C1R, C6, C7, CFP, and CFHR3 could be prognostic biomarkers for patients with HCC.

## Graphical abstract

C1R, C6, C7, CFP, and CFHR3 could be prognostic biomarkers for patients with HCC. Based on that, we developed a prognostic model for HCC patients—Riskscore = (−0.0053)*C6+(−0.0498)*C7+(−0.1045)*CFHR3.

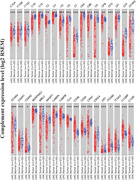

## 1 INTRODUCTION

The incidence of liver cancer has increased rapidly worldwide in recent years. Study shows that its morbidity has elevated to 6th in cancer, while it also ranks the third death cause of cancer.^1^ Hepatocellular carcinoma (HCC) represents approximately 90% of primary liver cancer.^2^ In the tides of immune therapy, patients with many other kinds of cancers are benefited from the novel therapy.^3^ However, the prognosis of patients with HCC is not optimistic.^4^, ^5^, ^6^ By deeper understanding the role of the immune system in hepatocellular carcinoma, we may discover novel prognostic biomarkers and advanced treatment.

The complement system is a fundamental branch of innate immunity of human body, containing more than 50 inherent components and membrane binding receptors and regulators. Three main pathways of complement activation depending on C3^7^ have been described: the classical pathway (CP), the alternative pathway (AP), and the lectin pathway. Other factors, such as proteases, kallikrein, plasmin, and factor XIIa, can generate complement activation products without C3.^8^ Thrombin, a member of the coagulation pathway, can locally generate C5a in C3‐deficient mice which are unable to generate the conventional C5 convertase.^9^

It is reported that the complement system has a complicated relationship with human cancer.^10^ On one hand, the complement activation may act as antitumor defense mechanism because of its participation in immune surveillance and complement‐dependent cytotoxicity. Rituximab, an anti‐CD20 antibody against malignant B cells, acts its antitumor effect by activating the classical complement pathway in vitro and in vivo studies.^11^, ^12^ On the other hand, it is now widely believed that the activation of the complement system may promote tumorigenesis and cancer progression by causing inflammation in the tumor microenvironment.^13^ In this situation, complement activation may not strengthen immune surveillance but, instead, cause immunosuppression by upregulating cytokines such as IL‐10, PDL‐1, CD46 in tumor cells as reported.^14^ Also, complement receptors, C3aR and C5aR, are considered to promote cancer cell proliferation in cancers, including, ovarian cancer,^15^ colon cancer,^16^ lung cancer,^17^, ^18^, ^19^ and so on. The effects of complement on HCC have also been explored but remain unclear. It is reported that Aristolochic acid I, which is considered to be hepatotoxic, promotes the invasion and migration of HCC cells by activating the C3a/C3aR complement system.^20^ Chen et al.^21^ reported downregulation of C3aR/C5aR inhibits cell proliferation and epithelial–mesenchymal transition (EMT) in HCC. Complement factor H, a negative regulator of the alternative pathway of the complement system, has been reported that its deficiency in mice caused spontaneous hepatic tumors.^22^ Recent studies show that enhanced CFH or CFHR (CFHR1 and CFHR3) levels either by gene therapies or by CFH reconstitution might lower tumor burden in HCC.^23^ However, previous studies have focused on particular complement members' effect on HCC. The role of other complement members remained unknown in the development and progression of HCC.

To address this, we thoroughly explored the expressions of the complement‐related genes and their correlation with prognosis and immune infiltration in patients with HCC via The Cancer Genome Atlas (TCGA) public and GEO database for the first time.

## 2 MATERIALS AND METHODS

### 2.1 Study patients

TCGA is a comprehensive database containing information on more than 30 kinds of human cancers, including mRNA sequencing, DNA sequencing, pathological data, and clinical pathological information.^24^ In this study, we downloaded clinic pathological information and mRNA expressions of 364 HCC patients from TCGA. Table 1 showed clinical data of all the 364 patients, including gender, age, weight, AFP, Child‐Pugh stage, adjacent tissue inflammation, cirrhosis, histologic grade, pathologic stage, and so on.

**Table 1:** Basic characteristics of 364 HCC patients

Variables	HCC patients (*N* = 364)
Gender (Male/female)	246/118
Age (years, mean ± *SD*)	59.67 ± 13.37
Weight (kg, median)	69 (40–172)
PLT (10e9/L, median)	211 (4–499000)
Albumin (g/L, median)	4 (0.2–5200)
Creatinine (mg/dl, median)	0.9 (0.4–12.4)
PT (s, median)	1.1 (0.8–36.4)
TB (μmol/L, median)	1.2 (0.2–2.1)
AFP (ng/ml, median)	15 (1–2035400)
*Child‐Pugh stage*	
A	*N* = 126
B	*N* = 21
C	*N* = 1
*Adjacent tissue inflammation*	
Non	*N* = 117
Mild	*N* = 97
Severe	*N* = 17
*Cirrhosis*	
Non‐cirrhosis	*N *= 74
Cirrhosis	*N* = 134
*Histologic grade*	
1	*N* = 55
2	*N* = 174
3	*N* = 118
4	*N* = 12
*Pathologic stage*	
1	*N* = 170
2	*N* = 83
3	*N* = 83
4	*N* = 4

Abbreviations: AFP, alpha fetoprotein; HCC, hepatocellular carcinoma; PLT, platelet; PT, prothrombin time; SD, standard deviation; TB, total bilirubin.

As there are only 50 normal liver samples in TCGA database, we also collected HCC data set GSE25097 from Gene Expression Omnibus (GEO, http://www.ncbi.nlm.nih.gov/geo/). This data set contains 268 HCC samples and 243 normal controlled samples from HCC patients.^25^ The data set was estimated thoroughly through the full text.

### 2.2 Inclusion and exclusion criteria

The inclusion criterion was as follows: (1) patients in the TCGA and GSE25097 databases were diagnosed with HCC. (2) There were clear criteria for the diagnosis and staging of HCC. (3) The study data provided OR (odds ratio)/HR (hazard ratio) and its 95% confidence interval (CI), or could be converted to OR and its 95% CI. The exclusion criterion was as follows: (1) Repeat recorded cases. (2) Data was incomplete.

### 2.3 Differential expression analysis

First, differences in mRNA expressions of the whole complement system between cancer samples and normal controlled samples were analyzed by TIMER2.TIMER2 (http://timer.cistrome.org/) is an online analysis tool that can not only analyze gene expression profiles in different kinds of cancers, but also provide an advanced algorithm to evaluate the abundance of tumor‐infiltrating immune cells by gene expression profiles based on TCGA database.^26^

Then, we used the pair‐controlled samples for HCC samples in GEPIA database and datasets GSE25097 to further verify the different mRNA expressions between HCC samples and their normal counterparts. Gene Expression Profiling Interactive Analysis (GEPIA, www.gepia.cancer-pku.cn) is a web server analyzing RNA sequencing expression data of tumors and normal samples from both TCGA and Genotypic Tissue Expression (GTEx) projects using standard processing pipelines.^27^

Next, UALCAN database was used to examine the relationship between mRNA expressions of the complement system and patients' clinicopathologic information, such as tumor stage and grade. UALCAN (http://ualcan.path.uab.edu), a web tool based on level 3 RNA‐seq and clinical data of 31 cancer types from TCGA database, can be used to explore the association of the transcriptional expression of a specific gene with cancer patients' clinicopathologic parameters.^28^

### 2.4 Survival analysis

In this study, we evaluated the prognostic value of mRNA expressions of the complement system in patients with HCC by calculating overall survival (OS), disease‐free survival (DFS), and progress‐free survival (PFS) using the Kaplan–Meier plotter (www.kmplot.com), which is a web tool for survival analysis by product‐limit method based on gene expression data and survival information of patients with different kinds of cancers based on TCGA database.^29^, ^30^ Information can be found on the Kaplan–Meier plotter web page, including number of patients, best cutoff values of mRNA expression, 95% CI, HR, and *p*‐value.

### 2.5 Mutation analysis

We used cBioPortal database to investigate the mutation rate of the whole complement system in HCC patients and to confirm its relation with the survival of HCC patients. cBioPortal, a database that includes data for putative copy‐number alterations (CNAs), mRNA expression *z*‐scores, and mutations, allows researchers to explore multidimensional cancer genomics data.^31^

### 2.6 PPI and enrichment analysis

We performed a protein–protein interaction (PPI) network analysis on the prognostic genes of the complement system to explore their interactions by STRINGS (www.string-db.org), an online analysis tool that collects, scores and integrates all publicly available sources to predict PPI between various proteins.^32^

LinkedOmics was used to evaluate both positively and negatively co‐expressed genes of the prognostic complement genes of the complement system in HCC patients. LinkedOmics database predicted co‐expressed genes of a specific gene by Spearman's correlation coefficient test using multi‐omics data for 32 cancer types and a total of 11,158 patients from TCGA project.^33^

Next, we used the list of co‐expressed genes to conduct Gene Ontology (GO) and Kyoto Encyclopedia of Genes and Genomes (KEGG) enrichment analysis to predict the function of these prognostic genes in HCC by Metascape (http://metascape.org), a free and well‐maintained online bioinformatics database for GO and KEGG enrichment analysis.^34^ In this study, we considered as significant only when *p* < .01, minimum count > 3, and enrichment factor > 1.5.

### 2.7 Immune infiltration analysis

We first used TIMER2 to detect the relationship between the mRNA expression of the whole complement system and tumor immune cells infiltration, including CD8+ and CD4+ T cells, macrophages, neutrophils, and dendritic cells (DCs), by spearsmans' correlation coefficients. Then, we examined the relationships between the prognostic complement genes and immune gene marker sets in HCC sample from TCGA using TIMER2. The gene markers of tumor‐infiltrating immune cells, including T cells, monocytes, tumor‐associated macrophages (TAMs), M1 and M2 macrophages, neutrophils, DCs, Tregs, and T cell exhaustion, were referenced from prior studies.^24^, ^29^, ^30^

### 2.8 Construction of the prognostic model

A prognostic model was constructed based on our results of the complement expressions and OS prognostic information in the cluster. The method of the least absolute shrinkage and selection operator (LASSO) regression algorithm is applied to find a model that can best explain the data but contains the fewest free parameters.

First, timeROC(v 0.4) analysis was performed to compare the predictive accuracy of each gene and score. Then, LASSO regression algorithm for feature selection, using tenfold cross‐validation. The K–M survival analysis with log‐rank test was also used to test the constructed model. All analytical methods above were performed using R software version 4.0.3.

### 2.9 Statistical analysis

The differential expression of complements was explored using the Wilcoxon test in TIMER2, ANOVA in GEPIA, and Students' *t*‐test in UALCAN and GSE25097. A fold change >2 or <0.5, or log2(FC) > 1 or <−1 with *p*‐values < .05 in both Gepia database and GSE25097 were considered statistically significant in mRNA expression in this study. The K–M curve diagrams of patients' survival were displayed as HR and log‐rank *p*‐values. In Spearman's correlation coefficients, we defined the strength of correlation as follows: 0.00–0.1, negligible correlation; 0.10–0.39, weak correlation; 0.40–0.69, moderate correlation; 0.70–0.89, strong correlation; and 0.90–1.0, very strong correlation. In this study, *p*‐values < .05 were considered statistically significant.

## 3 RESULTS

### 3.1 The differential expression of complement in hepatocellular carcinoma

**Figure 1:**
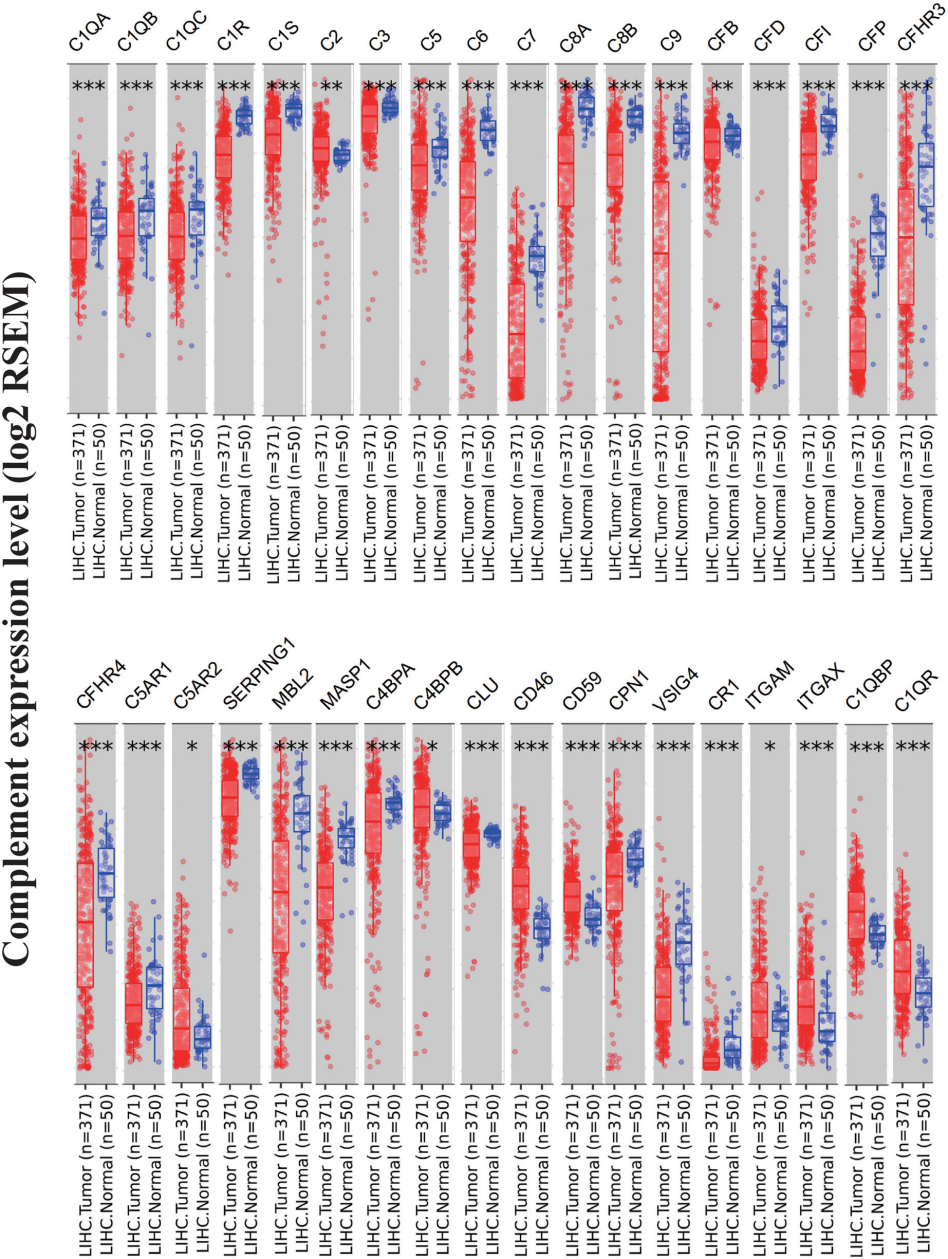
Transcriptional expression levels of complements in hepatocellular carcinoma in the TIMER2 database. ***p < .001, **p < .01, *p < .05

First, Table S1 showed the complement members evaluated in this study and their classification. Then, mRNA expressions of complement genes were explored via TIMER2, which gave us a general idea of the differences in complement gene expressions between HCC (*n* = 371) and normal tissues (*n* = 50) in patients (Table S2). Our results revealed that 27 complement genes, including C1QA, C1QB, C1QC, C1R, C1S, C3, C5, C6, C7, C8A, C8B, C9, CFB, CFD, CFI, CFP, CFHR3, CFHR4, C5AR1, SERPING1, MBL2, MASP1, C4BPA, CLU, CPN1, VSIG4, and CR1, were significantly downregulated in HCC tissues than in normal tissues (*p* < .05). In contrast, nine complement genes, including C2, C5AR2, C4BPB, CD46, CD59, ITGAM, ITGAX, C1QBP, and C1QR, were significantly upregulated in HCC tissues compared with normal samples (*p* < .05, Figure 1).

**Table 2:** The significant changes of complement expression in transcription level between HCC and normal liver tissues

Gene	Fold change (HCC vs. Normal)	*p*
C1QA	0.52	**
C1QB	0.80	**
C1QC	0.55	**
C1R	0.33	**
C1S	0.80	**
C2	1.07	**
C3	0.94	**
C5	0.82	**
C6	0.35	**
C7	0.15	**
C8A	0.61	**
C8B	0.62	**
C9	0.39	**
CFB	0.92	**
CFD	0.62	**
CFI	0.57	**
CFP	0.10	**
CFH	0.86	**
CFHR3	0.45	**
CFHR4	1.11	0.07
C5AR1	0.71	**
SERPING1	0.80	**
C5AR2	1.09	*
MASP1	0.28	**
MBL2	0.52	**
C4BPA	0.78	**
C4BPB	0.90	**
CLU	0.89	0.11
CPN1	0.61	**
VSIG4	0.78	**
CR1	0.51	**
ITGAM	1.10	0.22
ITGAX	1.16	0.06
CD93	1.09	0.09
CD46	1.24	**
CD59	0.98	0.44
C1QBP	0.80	**

Abbreviation: HCC, hepatocellular carcinoma.

* *p* < .05

** *p* < .01.

**Figure 2:**
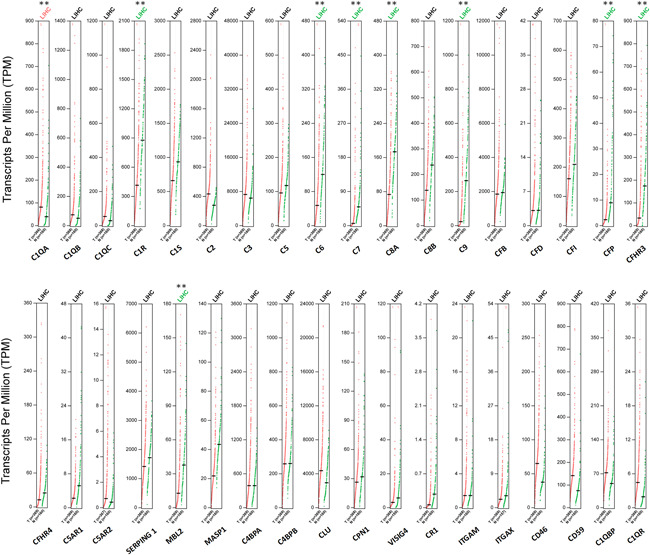
The mRNA expressions of complements in HCC tissues (GEPIA). HCC, hepatocellular carcinoma. ***p < .001, **p < .01, *p < .05

To further confirm the differential gene expressions between HCC samples and normal tissues, we used Gepia database and an independent data set (GSE25097), since they contained more paired normal liver samples. According to the result from Gepia database (Figure 2), the mRNA expressions of C1R, C6, C7, C8A, C9, CFP, CFHR3, and MBL2 were still significantly lower in HCC samples (*n* = 368) than in normal samples (*n* = 160), while the mRNA expression of C1QA was significantly higher in HCC samples (*n* = 368) than in normal samples (*n* = 160). The expression of the other 27 complements showed no difference between HCC samples and normal tissues, with a log_2_(FC) > 1 or <−1 and *p*‐value < .05. We then used data set (GSE25097) to confirm the mRNA expression of all the complement genes. Our result revealed that the mRNA expressions of C1R, C6, C7, C9, CFP, CFHR3, and MASP1 were significantly downregulated in HCC tissues (*n* = 268) compared with normal liver tissues (*n* = 243), with a fold change >2 or <0.5 and *p*‐value < .05 (Table 2), while the expression of the rest complement genes showed no difference between HCC samples and normal tissues.

To sum up, C1R, C6, C7, C9, CFP, and CFHR3 were significantly downregulated in all three HCC data sets. C8A, MASP1, and MBL2 were found downregulated in two HCC data sets. C1QA was found downregulated in TIMER2 database, but was found upregulated in Gepia database. In GSE25097, mRNA expression of C1QA was not significantly altered in HCC samples. So, we considered that it need further validation. Other complement genes, including C1QB, C1QC, C1S, C2, C3, C5, CFB, CFD, CFI, CFHR4, C5AR1, SERPING1, C4BPA, C4BPB, CLU, CPN1, VSIG4, CR1, ITGAM, ITGAX, CD46, CD59, and C1QBP transcription levels were significantly down‐ or upregulated in HCC samples according to TIMER2, while no difference was found in their transcription level according to Gepia and GSE25097 database. So, we considered the mRNA expression of these genes in HCC tissues and normal liver tissues was not clear and definite.

In general, our result showed that the mRNA expression of six complement genes, including C1R, C6, C7, C9, CFP, and CFHR3, were significantly lower in HCC tissues than their normal counterparts.

### 3.2 Prognosis analysis of complements in HCC

**Figure 3:**
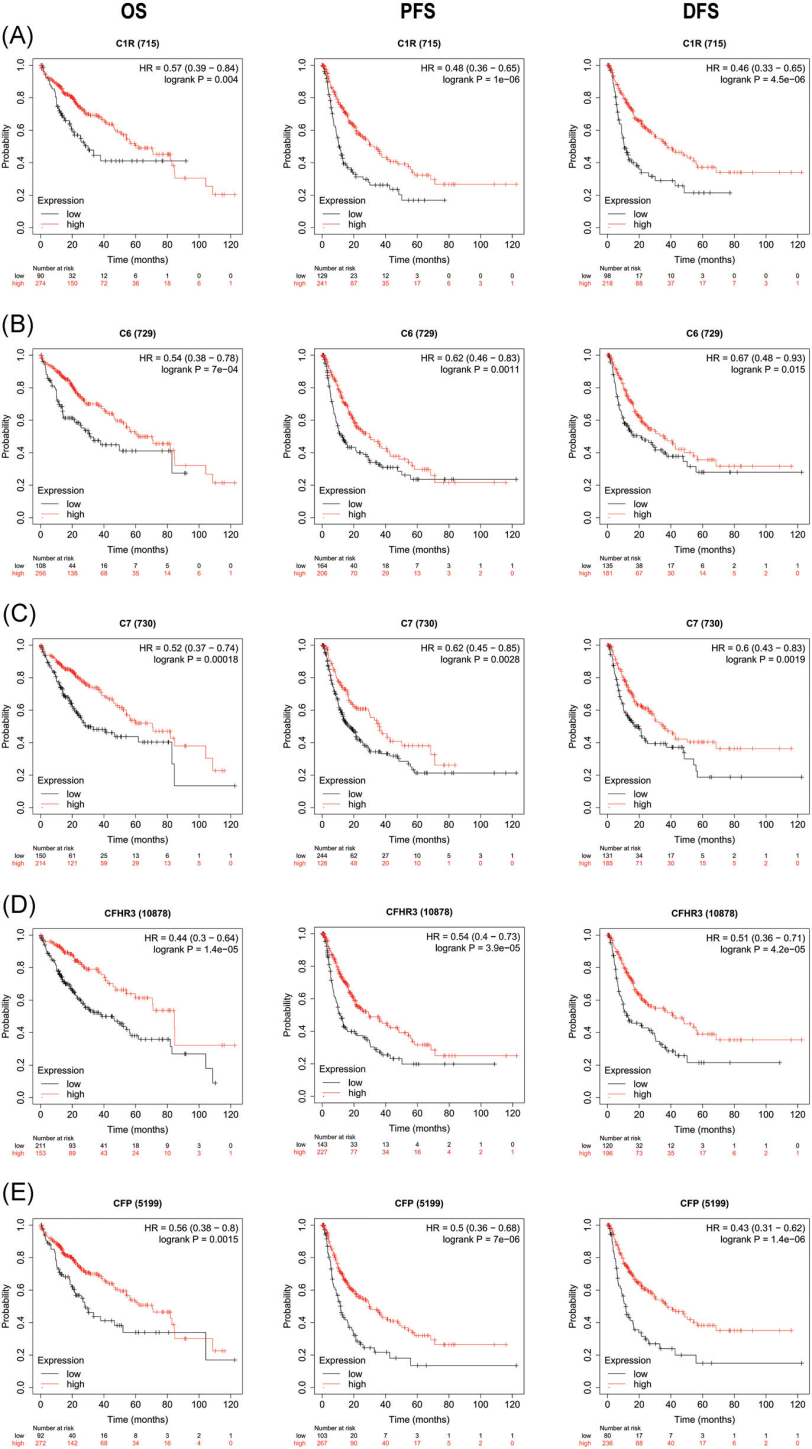
Prognostic value of mRNA expression of complement genes in patients with HCC (Kaplan–Meier Plotter). DFS, disease‐free survival; HCC, hepatocellular carcinoma; OS, overall survival; PFS, progress‐free survival

We analyzed the correlation between transcription levels of complement genes and patients' survival by Kaplan–Meier plotter database as survival analysis might indicate novel potential pathogenesis of HCC and promote further research. The results showed that higher mRNA expressions of 32 genes of the complements, namely C1R, C1S, C2, C3, C5, C6, C7, C8A, C8B, C8G, CFB, CFI, CFP, CFH, CFHR1, CFHR2, CFHR3, CFHR4, CFHR5, SERPING1, MASP2, MASP1, MBL2, C4BPA, C4BPB, VTN, CLU, CD59, CPN1, and CR2 were associated with longer OS in HCC patients while the higher mRNA expression of C5AR2 and ITGAM was associated with shorter OS in HCC patients (Table 3). To further evaluate the prognosis value of complement genes in HCC, we first explored the prognosis value of C1R, C6, C7, CFP, and CFHR3 via DFS and PFS, since their mRNA expressions were significantly downregulated in HCC tissues. We found that higher expressions of C1R, C6, C7, CFP, and CFHR3 were also associated with better DFS and PFS in HCC patients (Figure 3). Therefore, it was conceivable that higher mRNA expressions of C1R, C6, C7, CFP, and CFHR3 were independent biomarkers that predict a better prognosis in patients with HCC.

**Table 3:** Correlation of complement gene expression and overall survival in HCC patients via Kaplan–Meier plotter

Complement gene	Medium survival months		Hazard ratio	*p*
	Low expression cohort (months)	High expression cohort (months)		
C1QA	59.7	56.2	0.81	0.24
C1QB	46.6	70.5	0.77	0.15
C1QC	46.6	70.5	0.76	0.14
C1R	30	61.7	0.57	**
C1S	31	70.5	0.55	**
C2	37.8	70.5	0.56	**
C3	33.5	71	0.48	**
C4A	56.2	84.4	0.67	0.06
C5	33.5	70.5	0.49	**
C6	31	61.7	0.54	**
C7	28.3	70.5	0.52	**
C8A	45.7	70.5	0.59	**
C8B	45.7	84.4	0.47	**
C8G	27.9	70.5	0.50	**
C9	56.2	70.5	0.75	0.15
CFB	38.3	61.7	0.57	**
CFD	52	56.5	0.74	0.10
CFI	31	61.7	0.64	*
CFP	28.3	70.5	0.56	**
CFH	30	70.5	0.49	**
CFHR1	40.3	71	0.50	**
CFHR2	37.8	70.5	0.54	**
CFHR3	38.3	84.4	0.44	**
CFHR4	25.6	71	0.41	**
CFHR5	33.5	70.5	0.60	**
C3AR1	84.7	47.4	1.22	0.28
C5AR1	59.7	47.4	1.24	0.25
C5AR2	108.6	49.7	1.55	*
SERPING1	33.5	61.7	0.59	**
MASP1	38.3	71	0.47	**
MASP2	33.5	71	0.54	**
MBL2	54.1	71	0.63	*
C4BPA	33.5	81.9	0.53	**
C4BPB	33.5	70.5	0.57	**
VTN	33.5	71	0.49	**
CLU	33.5	70.5	0.55	**
CD46	54.1	61.7	0.76	0.13
CD55	82.9	52	1.22	0.31
CD59	33.5	61.7	0.65	*
CPN1	45.7	61.7	0.64	*
VSIG4	59.7	54.1	1.26	0.23
CR1	56.5	70.5	0.78	0.19
CR2	40.3	61.7	0.69	*
ITGAM	56.5	54.1	1.40	*
ITGAX	46.6	59.7	0.78	0.19
CD93	71	54.1	1.13	0.50
C1QBP	56.2	70.5	0.86	0.40

Abbreviation: HCC, hepatocellular carcinoma.

* *p* < .05

** *p* < .01.

Then, we further evaluated the prognostic value of the rest complement genes, whose mRNA expressions were not altered in HCC tissues, including C1S, C2, C3, C5, C8A, C8B, C8G, CFB, CFI, CFH, CFHR1, CFHR2, CFHR4, CFHR5, SERPING1, MASP1, MASP2, MBL2, C5AR2, C4BPA, C4BPB, VTN, CLU, CD59, CPN1, CR2, and ITGAM. According to our results shown in Tables 4 and 5, we found that lower expressions of C1S, C2, C3, C5, C8B, CFB, CFI, CFH, CFHR1, CFHR2, CFHR4, SERPING1, MASP2, MASP1, MBL2, C4BPA, C4BPB, VTN, CLU, CPN1, and CR2 were associated with shorter DFS and PFS in patients with HCC. However, lower expressions of C8A, C8G, CFHR5, and CD59 were only associated with shorter PFS in patients with HCC.

**Table 4:** Correlation of complement gene expression and disease‐free survival in HCC patients via Kaplan–Meier plotter

Complement gene	Medium survival months		Hazard ratio	*p*
	Low expression cohort (months)	high expression cohort (months)		
c1s	11.83	37.23	0.51	**
c2	18.3	37.67	0.57	**
c3	18.3	37.23	0.59	**
c5	13.27	40.97	0.51	**
C8A	21.23	30.1	0.74	0.09
C8B	16.6	36.1	0.63	**
C8G	23.97	33	0.83	0.31
CFB	12.87	40.97	0.52	**
CFI	23.97	37.23	0.67	*
CFH	15.07	36.1	0.64	*
CFHR1	21.3	55.87	0.48	**
CFHR2	18.87	42.87	0.53	**
CFHR4	10.5	37.67	0.51	**
CFHR5	25.87	36.1	0.69	0.05
SERPING1	13.27	36.1	0.6	**
MASP1	18.87	42.63	0.63	**
MASP2	13.27	34.4	0.6	**
C5AR2	37.67	25.13	1.33	0.10
MBL2	21.2	36.1	0.7	*
C4BPA	13.27	37.23	0.58	**
C4BPB	16.83	37.67	0.69	*
VTN	21.87	37.67	0.71	*
CLU	10.03	34.4	0.56	**
CD59	21.3	33	0.86	0.37
CPN1	15.07	36.1	0.62	*
CR2	21.47	37.67	0.7	*
ITGAM	21.87	34.4	0.81	0.22

Abbreviation: HCC, hepatocellular carcinoma.

* *p* < .05

** *p* < .01.

**Table 5:** Correlation of complement gene expression and progress‐free survival in HCC patients via Kaplan–Meier plotter

Complement gene	Medium survival months		Hazard ratio	*p*
	Low expression cohort (months)	high expression cohort (months)		
c1s	11.97	30.4	0.55	**
c2	15.07	33	0.56	**
c3	11.83	29.73	0.55	**
c5	14.33	42.87	0.51	**
C8A	11.9	25.87	0.68	*
C8B	13.27	29.77	0.65	**
C8G	13.13	29.3	0.68	*
CFB	10.67	33	0.51	**
CFI	15.07	30.4	0.64	**
CFH	11.9	30.1	0.60	**
CFHR1	12.87	30.1	0.57	**
CFHR2	13.27	36.27	0.56	**
CFHR4	9.77	29.77	0.48	**
CFHR5	17.9	33	0.7	*
SERPING1	11.83	30.4	0.58	**
MASP1	15.97	36.1	0.61	**
MASP2	11.83	29.77	0.58	**
C5AR2	27.6	21.3	1.32	0.07
MBL2	14.33	29.3	0.72	*
C4BPA	10.27	30.1	0.54	**
C4BPB	15.07	33	0.66	**
VTN	13.27	29.17	0.61	**
CLU	13.13	30.4	0.57	**
CD59	16.83	30.1	0.73	*
CPN1	13.83	27.6	0.7	*
CR2	13.83	25.87	0.71	*
ITGAM	25.87	19.93	1.26	0.2

Abbreviation: HCC, hepatocellular carcinoma.

* *p* < .05

** *p* < .01.

### 3.3 Relationship between mRNA levels of complement genes and the clinicopathological parameters of HCC patients

**Figure 4:**
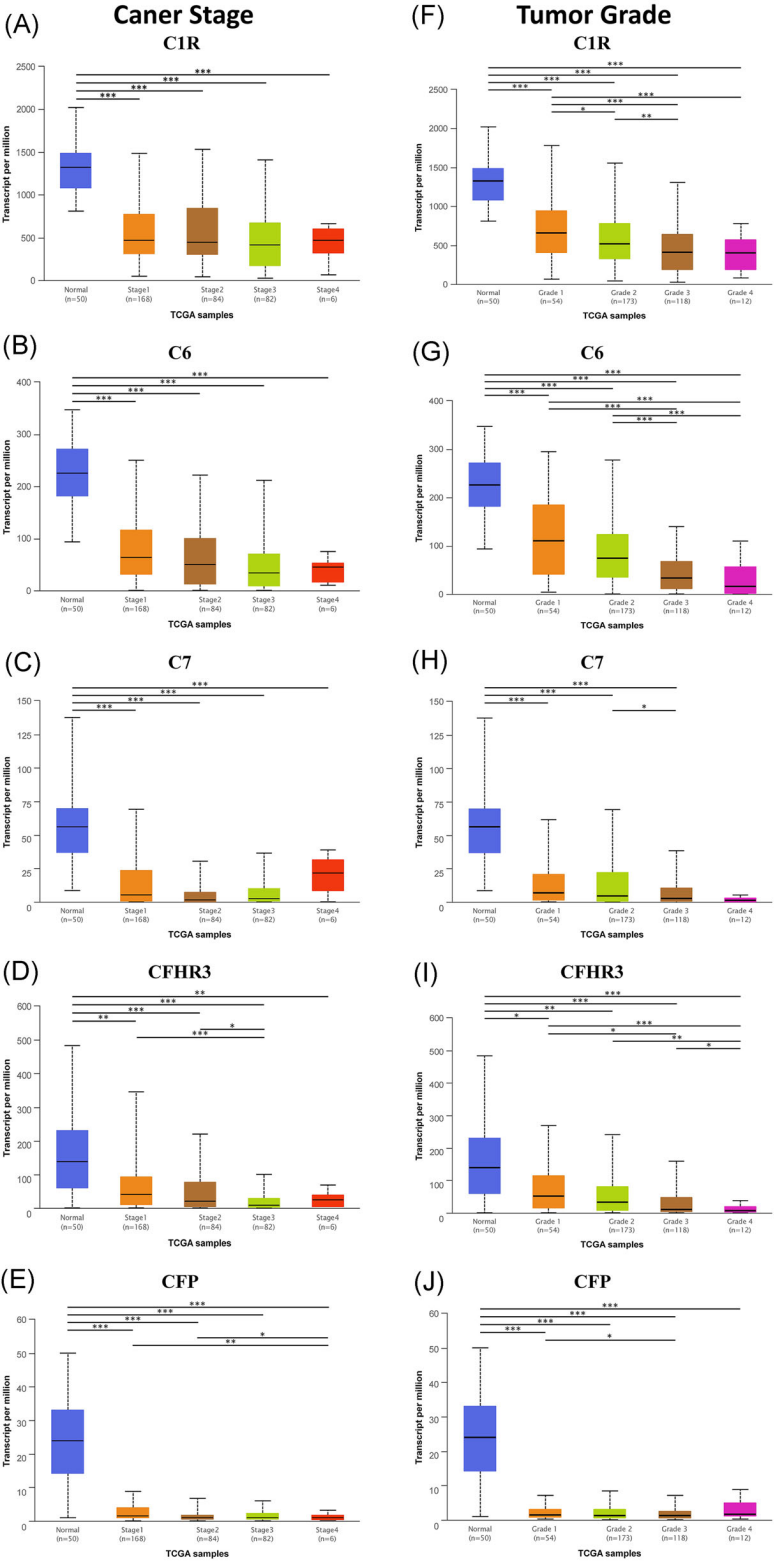
Relationship between mRNA expression of complement genes and individual cancer stages (A–E) and tumor grades (F–J) of HCC patients (Ualcan). HCC, hepatocellular carcinoma. ***p < .001, **p < .01, *p < .05

We further studied the relationship between mRNA expression levels of C1R, C6, C7, CFP, CFHR3, and tumor stages in HCC patients by using the UALCAN database since these genes were considered to have prognostic value. The results showed that the expression levels of C1R, C6, and CFHR3 were correlated with the tumor stage of HCC patients, while the expression levels of C7 and CFP were not correlated with the tumor stage of HCC patients. As was shown in Figure 4A–E, patients who were in more advanced cancer stage tended to have less mRNA expression of C1R, C6, and CFHR3. Although the lowest mRNA expression of C1R, C6, and CFHR3 was found in stage 3, it might result from the fact that there were only six patients at stage 4.

Similarly, we studied the relationship between mRNA expression levels of the five prognostic complement genes and tumor grade in HCC patients. The results showed that the mRNA expression levels of C1R, C6, C7, and CFHR3 were correlated with the tumor grade of HCC patients, while only CFP was not correlated with the tumor grade of HCC patients (Figure 4F–J). As tumor grades increased, the mRNA expressions of C1R, C6, C7, and CFHR3 tended to be lower. The lowest mRNA expressions level of C1R, C6, C7, and CFHR3 were all found in patients at grade 4.

Next, we examined the mRNA expressions of C1S, C2, C3, C5, C8B, CFB, CFI, CFH, CFHR1, CFHR2, CFHR4, SERPING1, MASP2, MASP1, MBL2, C4BPA, C4BPB, VTN, CLU, CPN1, and CR2 as they were also associated with OS, DFS, and PFS with HCC patients though their mRNA expressions were not altered in HCC tissues according to our results. The result showed that the lower mRNA expressions of C3, C5, CFB, and CLU were correlated with more advanced cancer stage, while the lower mRNA expressions of C1S, C8B, CFI, MBL2, and C4BPA were correlated with higher tumor grade in HCC patients (Table 6). However, no gene was found correlated with both cancer stage and tumor grade in this set.

**Table 6:** Relationship between mRNA expression of complement genes and cancer stages or tumor grades of HCC patients

	Cancer stage (medium expression)					Tumor grade (medium expression)				
Gene	Normal	Stage 1	Stage 2	Stage 3	Stage 4	normal	Grade 1	Grade 2	Grade 3	Grade 4
C1S	1581.2	809.9	771.9	815.5	653.1	1581.2	974.5	889.2	714.4	497.8
C2	418.9	463.3	503.9	393.8	636.4	418.9	443.6	465.9	474.7	385.8
C3	6491.2	5625.1	4859.5	4027.3	5877.9	6491.2	5568.3	5729.3	4393.1	3918.8
C5	134.6	102.3	91.5	83.7	90.4	134.6	116.2	91.1	90.3	49
C8B	384.2	179.8	145.4	152.2	152.2	384.2	245.3	186.9	104.1	70.8
CFB	1971.7	1668.2	1474.9	1322.1	1239.2	1971.7	1945.9	1615.5	1503.1	1146.5
CFI	339.4	183.6	181.6	173.4	159.5	339.4	266.6	194.4	140.4	134.9
CFH	604.3	564.4	544.5	459.6	646.9	604.3	583.7	563.2	463.7	306.6
CFHR1	794.1	721.2	672.5	457.9	785.2	794.1	710.2	723.4	590.9	262.3
CFHR2	558.5	554.2	566.2	335.6	464.5	558.5	600.4	582.5	400.5	299.9
CFHR4	33.2	14.1	11.4	3.1	12.2	33.2	29.5	13.7	6.6	1.2
SERPING1	2903.2	1736.6	1638.4	1136.1	2203.6	2903.2	2026.7	1980.8	1288.9	1030.9
MASP1	55.8	20.5	17.8	23.5	22.6	55.8	27.6	21.1	19.1	10.1
MASP2	264.8	262.3	255.3	156.5	265.9	264.8	298.9	280.2	184.6	175.7
MBL2	62.0	18.9	9.5	13.2	18.2	62.0	23.6	16.4	13.53	6.87
C4BPA	752.4	463	539.1	231.9	438.4	752.4	650.2	527.3	336.9	168.5
C4BPB	262.6	304.2	317.3	242.7	385.9	262.6	298.2	312.7	277.8	194.3
VTN	5040.8	5295.1	4398.8	4309.3	5178.3	5040.8	5099.9	5136.7	4237.9	2852.9
CLU	2302.5	1761.1	1415.5	1249.8	1259.2	2302.5	1668.8	1519.5	1543	1400.2
CPN1	44.4	32.9	28.4	29.3	32.2	44.4	32.7	31.6	28.2	41.7
CR2	0.0	0	0	0	0	0	0	0	0	0

Abbreviation: HCC, hepatocellular carcinoma.

### 3.4 Genetic mutation in complement system and their association with OS and DFS in patients with HCC

As most of the complement components are produced in liver, we then analyzed the mutation of all the complement genes in HCC samples by cBioPortal database.

**Figure 5:**
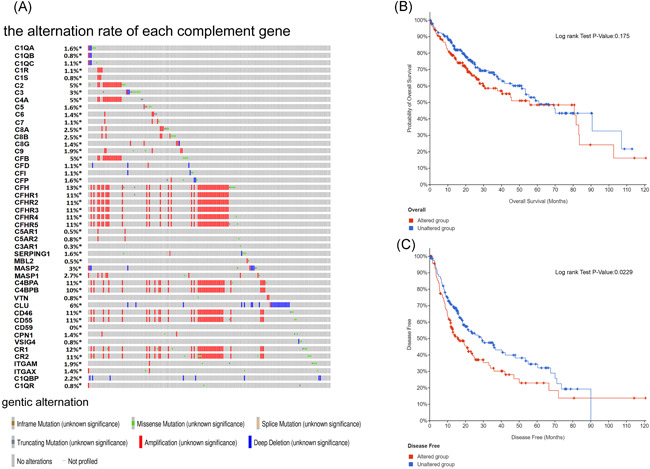
Genetic mutations of complement genes and their association with OS and DFS in patients with HCC (cBioPortal). (A) Mutation rate of complement genes in HCC samples. (B) The association between genetic alterations and OS in HCC patients. (C) The association between genetic alterations and DFS in HCC patients. DFS, disease‐free survival; HCC, hepatocellular carcinoma; OS, overall survival

As shown in Figure 5A, the mutation rate of each of the complement genes was relatively low in HCC patients. Among all the genes, CFH topped the mutation rate (13%), followed by CR1 (12%), CR2 (11%), C4BPA (11%), CFHR1 (11%), CFHR2 (11%), CFHR3 (11%), CFHR4 (11%), CFHR5 (11%), CD46 (11%), CD55 (11%), and C4BPB (10%). The mutation rate of the rest complement genes was less than 10%. In all the 364 HCC patients, no mutation was found in CD59 (0%). Furthermore, we found that the majority of the mutation was amplification. Then, we examined the association between genetic alteration in complement genes and the survival of HCC patients, including OS and DFS. According to the Kaplan–Meier plot (Figure 5B,C), the genetic alteration of the complement genes might have correlation with poorer DFS in HCC patients (*p* < .05) but did not affect OS in HCC patients.

### 3.5 PPI and functional enrichment analysis of complements in HCC patients

**Figure 6:**
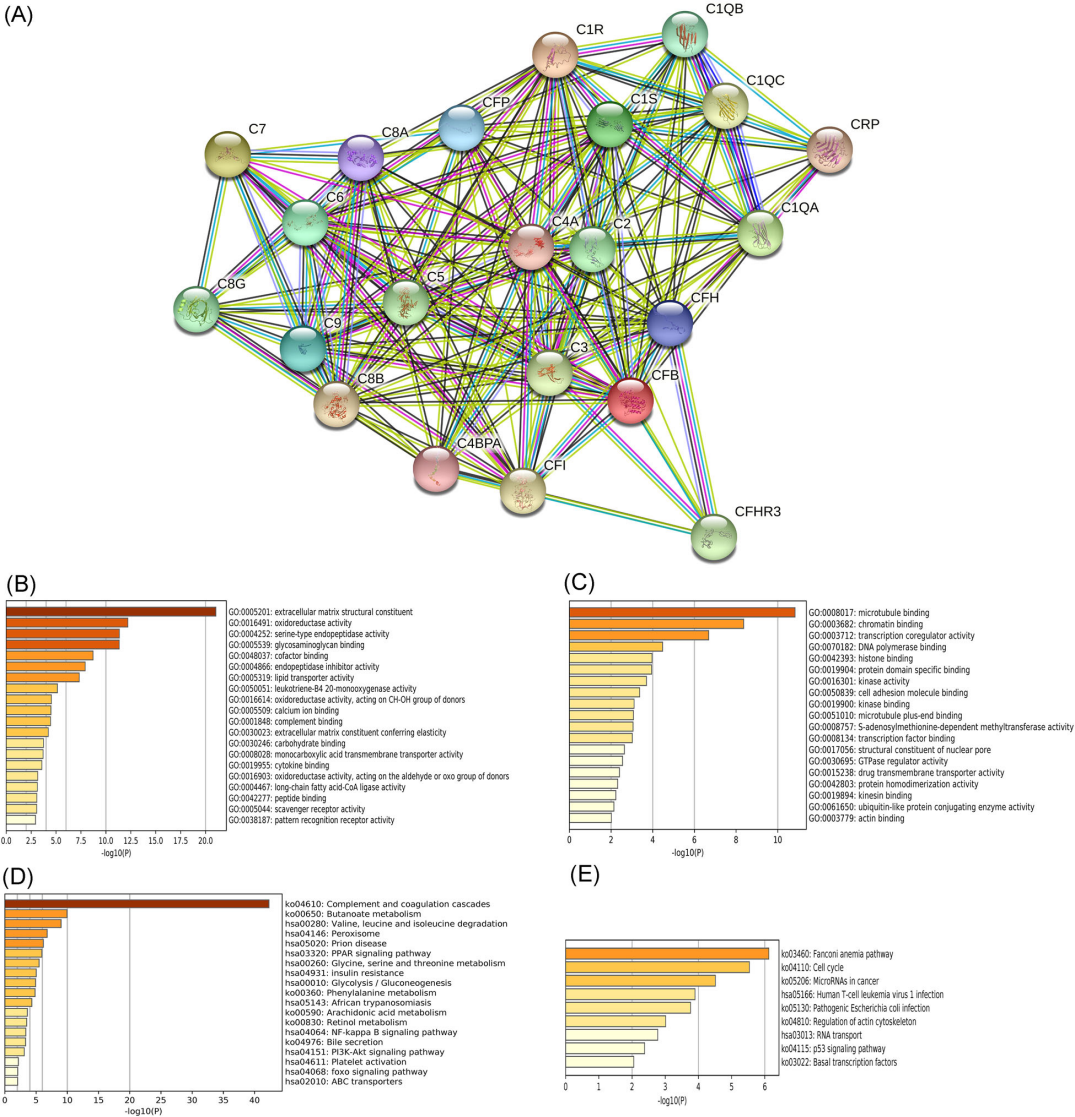
Protein–protein interaction (PPI) and functional enrichment analysis of five prognostic complement genes in patients with HCC (STRING and Metascape). (A) PPI of C1R, C6, C7, CFP, and CFHR3. (B) gene ontology (GO) of positively co‐expressed genes of C1R, C6, C7, CFP, and CFHR3, colored by *p*‐value. (C) GO of negatively co‐expressed genes of C1R, C6, C7, CFP, and CFHR3, colored by *p*‐value. (D) Kyoto Encyclopedia of Genes and Genomes (KEGG) of positively co‐expressed genes of C1R, C6, C7, CFP, and CFHR3, colored by *p*‐value. (E) KEGG of negatively co‐expressed genes of C1R, C6, C7, CFP, and CFHR3, colored by *p*‐value. HCC, hepatocellular carcinoma

Then, we used String database to build a PPI network between the five complements genes found above, including C1R, C6, C7, CFP, and CFHR3, as they were not only downregulated in HCC, but also correlated with the prognosis of patients with HCC. We found that these genes generally interacted with other complement genes to moderate the immune system and hormone system (Figure 6A). There were little information about these genes interact with other functions.

So, we calculated co‐expressed genes of these five complement genes in HCC samples from TCGA database. Table S3 showed each of 5 complement genes and their 50 most co‐expressed genes both negatively and positively by Spearman's analysis.

Next, we conducted GO and KEGG pathway enrichment analysis by these co‐expressed genes to further predict the function of the five prognostic complement genes in HCC patients. As shown in Figure 6B,C, molecular function such as GO: 0005201 (extracellular matrix structural constituent), GO: 0016491 (oxidoreductase activity), GO: 0004252 (serine‐type endopeptidase activity), and GO: 0005539 (glycosaminoglycan binding) were positively correlated with the expression of these 5 complement genes, while GO: 0008016 (microtubule binding), GO: 0003682 (chromatin binding), GO: 0003712 (transcription coregulator activity), and GO: 0070182 (DNA polymerase binding) were negatively correlated with the expression of these genes. Furthermore, we conducted KEGG analysis (Figure 6D,E), 19 pathways including hsa 03320: PPAR signaling pathway, hsa 04064: NF‐kappa B signaling pathway, and hsa 04151: PI3K‐Akt signaling pathway, were associated with overexpression of the five complement genes, while nine pathways including KO 03460: Fanconi anemia pathway, KO 05206: MicroRNAs in cancer, and KO 04115: P53 signaling pathway were associated with down‐expression of these genes. All these findings showed these five complement genes might correlate with confirmed pathways of HCC according to previous studies. Also, the result also showed that these five complement genes might correlate with biological function in pathways other than the complement or the immune system, indicating further research would be needed.

### 3.6 Correlation between complement expression and immune marker sets

Given the relationship of complements with the immune system, we further studied the correlations between complement genes and marker genes of tumor‐infiltrating immune cells, including T cells, TAMs, M1 and M2 macrophages, monocytes, T cell exhaustion, Tregs, and DCs, from HCC tissues.

**Figure 7:**
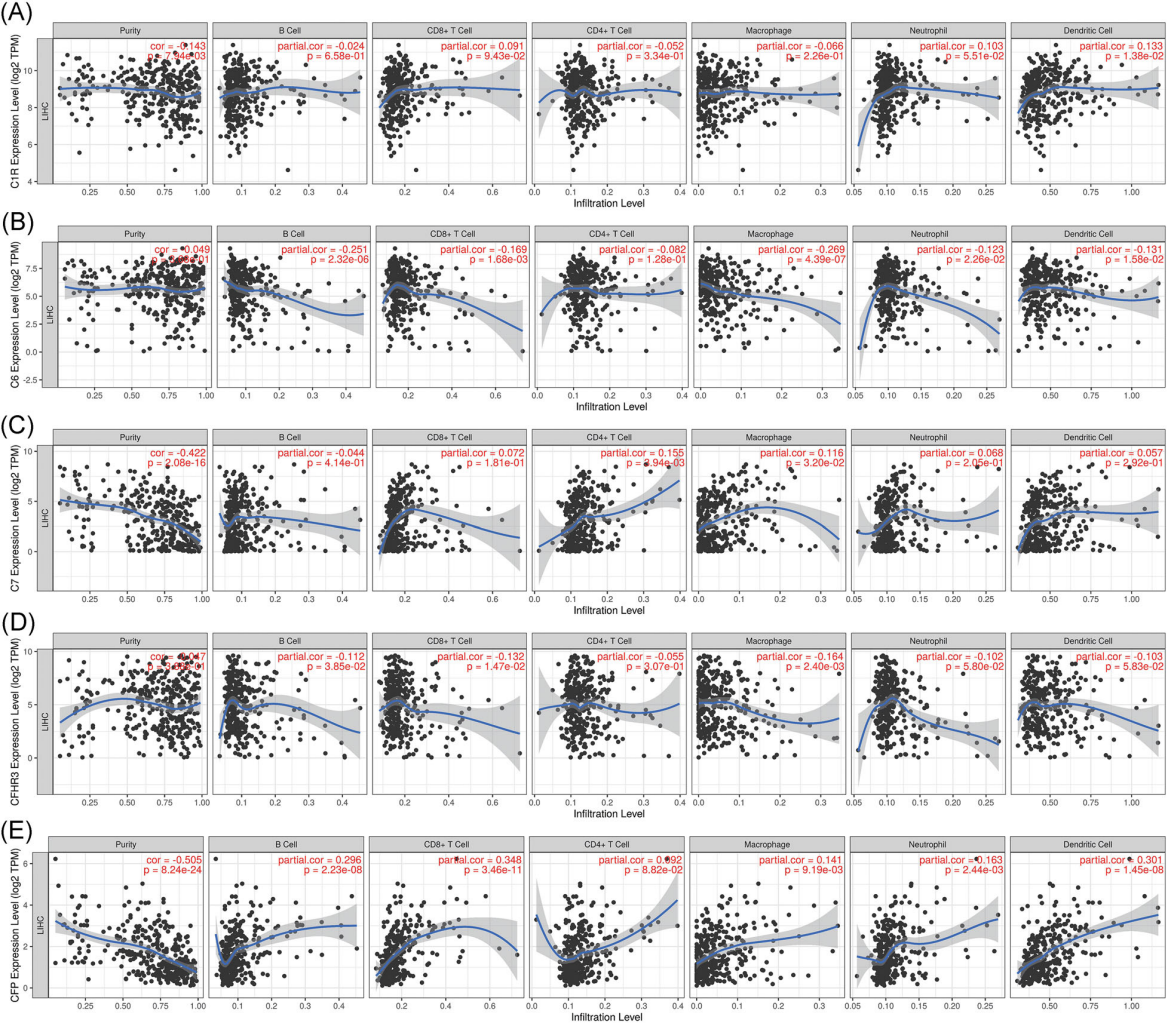
Correlation of C1R, C6, C7, CFP, and CFHR3 expression with immune infiltration levels in HCC. HCC, hepatocellular carcinoma

First, we examined C1R, C6, C7, CFP, and CFHR3 as they were considered to have prognosis values in HCC patients. Among them, we found only CFP had a statistical correlation with the immune cells in HCC samples. The mRNA expression of CFP had a weak positive correlation with CD8+ T cells and DCs (Figure 7).

Next, by correlation analysis adjusted by purity, we found that CFP expression was positively relevant to most sets of immune marker genes, such as those of CD8+ T cells (CD8A and CD8B), T cells (CD3E and CD2), B cells (CD79A), M2 macrophages (CD163), neutrophils (CCR7), DCs (CD1C, HLA‐DPA1, HLA‐DPB1, HLA‐DQB1, and HLA‐DRA), Th1 cells (IFNG and TBX21), Th2 cells (GATA3), and T cell exhaustion (GZMB, LAG3, and PDCD1; Table 7). In addition, the mRNA expression of C7 had a positive correlation with several sets of immune marker genes, such as TAMs (CCL2), M1 macrophages (NOS2 and PTGS2), and DCs (CD1C), while the mRNA expression of C6 had a negative correlation with TGFB1, an immune marker of Treg and PDCD1, an immune marker of T cell exhaustion. The rest two complement genes (C1R and CFHR3) had no correlation with the immune marker genes. Therefore, we believed the expression of CFP was correlated with tumor immune cells in HCC patients (Table 7).

**Table 7:** Correlation analysis between C1R, C6, C7, CFP, CFHR3, and relate immune genes markers dependently in HCC via TIMER

	CFP		C1R		C6		C7		CFHR3	
	Cor	*p*	Cor	*p*	Cor	*p*	Cor	*p*	Cor	*p*
*CD8+ T cell*										
CD8A	0.45	**	0.11	*	−0.10	0.07	0.08	0.13	−0.01	0.87
CD8B	0.42	**	0.07	0.21	−0.19	**	−0.01	0.81	−0.09	0.10
*T cell (general)*										
CD3D	0.31	**	−0.02	0.76	−0.28	**	−0.09	0.11	−0.11	*
CD3E	0.45	**	0.08	0.16	−0.18	**	0.08	0.16	0.00	0.96
CD2	0.43	**	0.07	0.20	−0.20	**	0.06	0.30	−0.00	0.99
*B cell*										
CD19	0.30	**	−0.04	0.44	−0.20	**	0.07	0.19	−0.06	0.30
CD79A	0.48	**	0.00	0.98	−0.21	**	0.12	*	−0.06	0.26
*Monocyte*										
CD86	0.30	**	0.07	0.23	−0.17	**	0.01	0.86	−0.10	0.06
CSF1R	0.33	**	0.16	**	−0.08	0.13	0.09	0.11	−0.04	0.49
*TAM*										
CCL2	0.20	**	0.13	*	0.02	0.73	0.40	**	0.03	0.59
CD68	0.10	0.05	0.01	0.90	−0.12	*	−0.05	0.36	−0.05	0.32
IL10	0.21	**	0.02	0.72	−0.11	*	−0.06	0.29	−0.13	*
*M1 macrophage*										
NOS2	0.10	0.07	0.13	*	0.12	*	0.47	**	0.28	**
IRF5	−0.09	0.15	−0.12	*	−0.15	**	0.11	*	−0.14	**
PTGS2	0.14	*	0.12	*	0.01	0.83	0.46	**	0.16	**
*M2 macrophage*										
CD163	0.37	**	0.28	**	0.13	*	0.17	**	0.12	*
VSIG4	0.17	**	0.29	**	0.08	0.14	0.16	**	0.09	0.09
MS4A4A	0.25	**	0.26	**	0.07	0.18	0.13	*	0.08	0.13
*Netrophil*										
CEACAM8	0.03	0.55	−0.03	0.55	−0.06	0.27	−0.05	0.38	−0.07	0.22
ITGAM	−0.03	0.55	0.17	**	−0.10	0.06	−0.04	0.42	−0.09	0.09
CCR7	0.42	**	0.10	0.06	−0.10	0.07	0.25	**	0.08	0.13
*Natural killer cell*										
KIR2DL1	0.08	0.13	0.12	*	0.06	0.28	−0.05	0.33	0.02	0.71
KIR2DL3	0.20	**	0.02	0.73	−0.04	0.49	−0.09	0.10	−0.10	0.06
KIR2DL4	0.21	**	0.16	**	−0.10	0.07	−0.13	*	−0.06	0.30
KIR2DS4	0.21	**	0.15	**	0.01	0.80	−0.01	0.80	0.03	0.60
KIR3DL1	0.20	**	0.11	*	0.12	*	−0.00	0.98	0.01	0.81
KIR3DL2	0.30	**	0.05	0.38	−0.06	0.26	0.05	0.35	−0.03	0.53
KIR3DL3	0.09	0.08	−0.01	0.78	−0.08	0.16	−0.05	0.37	−0.03	0.59
*Dendritic cell*										
CD1C	0.45	**	0.01	0.89	−0.07	0.20	0.32	**	0.06	0.24
HLA‐DPA1	0.37	**	0.22	**	−0.01	0.84	0.13	*	0.01	0.92
HLA‐DPB1	0.41	**	0.20	**	−0.09	0.09	0.11	*	−0.01	0.79
HLA‐DQB1	0.37	**	0.14	**	−0.09	0.10	0.02	0.67	−0.02	0.72
HLA‐DRA	0.34	**	0.23	**	−0.03	0.52	0.10	0.08	0.04	0.49
ITGAX	0.19	**	0.00	1	−0.19	**	−0.02	0.67	−0.09	0.10
NRP1	0.11	*	−0.04	0.42	−0.09	0.10	0.18	**	−0.19	0.00
*Th1 cell*										
IFNG	0.30	**	−0.01	0.87	−0.17	**	−0.13	*	−0.07	0.18
STAT1	0.22	**	0.04	0.44	−0.17	**	0.08	0.14	−0.13	*
STAT4	0.18	**	0.13	*	−0.07	0.22	0.16	**	0.06	0.29
TBX21	0.51	**	0.14	*	−0.03	0.54	0.07	0.17	0.06	0.28
TNF	0.24	**	0.02	0.77	−0.16	**	−0.04	0.44	−0.04	0.48
*Th2 cell*										
GATA3	0.31	**	0.08	0.14	−0.13	*	0.18	**	−0.04	0.43
IL13	0.05	0.34	0.06	0.31	0.06	0.26	0.05	0.39	0.02	0.74
STAT5A	0.21	**	0.06	0.24	−0.15	**	0.08	0.13	−0.17	**
STAT6	−0.01	0.83	0.14	*	0.08	0.13	0.17	**	−0.02	0.78
*Tfh cell*										
BCL6	0.08	0.16	0.00	0.97	−0.05	0.33	−0.05	0.37	−0.14	*
IL21	0.17	**	0.00	0.93	−0.14	*	−0.15	**	−0.11	0.05
*Th17 cell*										
IL17A	0.06	0.26	−0.05	0.40	−0.04	0.42	0.02	0.72	−0.02	0.68
STAT3	−0.13	*	0.29	**	0.16	**	0.18	**	0.04	0.51
*Treg*										
CCR8	0.11	*	−0.01	0.82	−0.11	*	0.10	0.08	−0.02	0.77
FOXP3	0.13	*	0.19	**	0.16	**	0.07	0.19	0.10	0.07
STAT5B	0.06	0.31	0.04	0.51	0.05	0.33	0.19	**	−0.07	0.17
TGFB1	0.17	**	−0.17	**	−0.36	**	0.19	**	−0.21	**
*T cell exhaustion*										
CTLA4	0.29	**	−0.07	0.23	−0.36	**	−0.21	**	−0.12	*
GZMB	0.50	**	0.06	0.23	−0.12	*	−0.11	*	−0.08	0.16
HAVCR2	0.19	**	0.05	0.34	−0.19	**	−0.04	0.50	−0.11	*
LAG3	0.39	**	0.02	0.67	−0.21	**	−0.10	0.06	−0.12	*
PDCD1	0.32	**	−0.06	0.24	−0.35	**	−0.10	0.08	−0.14	*

Abbreviation: HCC, hepatocellular carcinoma.

* *p* < .05

** *p* < .01.

Also, we examined the correlation between mRNA expressions of the complement genes other than these five genes and marker genes of tumor‐infiltrating immune cells. We found that the mRNA expression of C1QA, C1QB, C1QC, C5AR1, C3AR1, C1QR (CD93), CR1, CR2, CR3 (ITGAM), and CR4 (ITGAX) were positively associated with almost all kinds of tumor immune cells, including CD8+ T cells, CD4+ T cells, B cells, macrophages, monocytes and DCs (Table 8). Moreover, CFD showed a positive correlation with B cells, CD8+ T cells, macrophages, and DCs. Meanwhile, MASP2 showed a negative correlation with B cells, macrophages, and DCs (Table 8). Although these genes did not present prognostic value, further studies would be needed since their mRNA expressions were correlated with tumor immune cells might affect HCC.

**Table 8:** Correlation analysis between complement genes and relate immune cells in HCC via TIMER

	B cell		CD8+ T cell		CD4+ T cell		Macrophage		Netrophil		Dendritic cell	
Gene names	Cor	*p*	Cor	*p*	Cor	*p*	Cor	*p*	Cor	*p*	Cor	*p*
C1QA	0.52	**	0.62	**	0.20	**	0.58	**	0.42	**	0.68	**
C1QB	0.57	**	0.63	**	0.25	**	0.58	**	0.48	**	0.73	**
C1QC	0.59	**	0.61	**	0.33	**	0.64	**	0.64	**	0.75	**
C1R	−0.02	0.66	0.09	0.09	−0.05	0.33	−0.07	0.23	0.10	0.06	0.13	*
C1S	−0.06	0.25	−0.02	0.75	−0.07	0.21	−0.14	**	0.08	0.16	0.01	0.80
C2	0.03	0.56	−0.05	0.32	0.07	0.23	−0.03	0.54	−0.04	0.51	0.02	0.66
C3	−0.1	0.42	−0.16	**	0.01	0.91	−0.17	**	−0.12	*	−0.12	*
C4A	−0.09	0.10	−0.03	0.59	−0.08	0.12	−0.11	*	−0.07	0.19	0.05	0.36
C5	0.10	0.08	0.07	0.19	0.14	*	0.07	0.18	0.16	**	0.15	**
C6	−0.25	**	−0.17	**	−0.08	0.13	−0.27	**	−0.12	*	−0.13	*
C7	−0.04	0.41	0.07	0.18	0.16	**	0.12	*	0.07	0.21	0.06	0.29
C8A	−0.33	**	−0.18	**	−0.23	**	−0.30	**	−0.11	*	−0.17	**
C8B	−0.19	**	−0.08	0.13	−0.09	*	−0.18	**	−0.04	0.43	−0.05	0.33
C8G	−0.21	**	−0.19	**	−0.25	**	−0.30	**	−0.17	**	−0.27	**
C9	−0.04	0.43	−0.02	0.72	−0.03	0.61	−0.08	0.13	−0.01	0.87	0.089	0.10
CFB	−0.09	0.11	−0.06	0.25	−0.08	0.13	−0.21	**	−0.07	0.22	0	0.99
CFD	0.39	**	0.37	**	0.28	**	0.43	**	0.29	**	0.44	**
CFI	−0.13	*	−0.07	0.19	0.00	0.99	−0.12	*	0.04	0.49	0.01	0.81
CFP	0.30	**	0.35	**	0.09	8.08	0.14	**	0.16	**	0.30	**
CFHR1	−0.15	**	−0.14	*	−0.07	0.22	−0.16	**	−0.07	0.17	−0.17	**
CFHR2	−0.21	**	−0.17	**	−0.15	**	−0.29	**	−0.19	**	−0.22	**
CFHR3	−0.11	*	−0.13	*	−0.06	0.31	−0.16	**	−0.10	0.06	−0.10	0.06
CFHR4	−0.23	**	−0.24	**	−0.10	0.07	−0.33	**	−0.21	**	−0.22	**
CFHR5	−0.15	**	−0.17	**	0.01	0.83	−0.16	**	−0.05	0.31	−0.13	*
C5AR1	0.38	**	0.36	**	0.28	**	0.50	**	0.58	**	0.56	**
C5AR2	0.02	0.66	0.01	0.93	0.15	**	0.17	**	0.26	**	0.04	0.52
C3AR1	0.53	**	0.56	**	0.38	**	0.70	**	0.57	**	0.74	**
SERPING1	−0.04	**	−0.10	0.06	−0.13	*	−0.23	**	−0.12	*	−0.08	0.15
MASP2	−0.35	**	−0.27	**	−0.27	**	−0.41	**	−0.24	**	−0.31	**
MASP1	−0.12	*	−0.12	*	0	1.00	0.01	0.93	0.07	0.18	−0.12	*
MBL2	−0.20	**	−0.22	**	−0.06	0.29	−0.20	**	−0.14	*	−0.18	**
C4BPA	−0.16	**	−0.21	**	−0.07	0.18	−0.23	**	−0.09	0.08	−0.16	**
C4BPB	−0.20	**	−0.21	**	−0.20	**	−0.31	**	−0.25	**	−0.24	**
VTN	−0.03	0.64	−0.11	*	0.01	0.80	−0.11	0.04	−0.15	**	−0.10	0.06
CLU	−0.08	0.14	0.09	0.10	−0.07	0.21	−0.07	0.22	0.00	0.95	0.02	0.77
CD46	0.13	*	0.06	0.30	0.29	**	0.26	**	0.32	**	0.17	**
CD55	−0.08	0.14	0.01	0.82	0.10	0.07	0.11	*	0.25	**	0.06	0.24
CD59	0.14	*	0.18	**	0.43	**	0.39	**	0.38	**	0.28	**
CPN1	−0.02	0.69	−0.12	*	−0.05	0.38	−0.1	0.06	−0.12	*	−0.07	0.18
VSIG4	−0.02	0.69	−0.12	*	−0.05	0.38	−0.1	0.06	−0.12	*	−0.07	0.02
CR1	0.46	**	0.49	**	0.30	**	0.48	**	0.47	**	0.60	**
CR2	0.34	**	0.28	**	0.36	**	0.32	**	0.29	**	0.33	**
ITGAM	0.40	**	0.39	**	0.40	**	0.53	**	0.52	**	0.55	**
ITGAX	0.59	**	0.46	**	0.50	**	0.60	**	0.55	**	0.67	**
C1QR	0.25	**	0.30	**	0.47	**	0.50	**	0.53	**	0.48	**
C1QBP	0.23	**	0.20	**	0.08	0.13	0.20	**	0.20	**	0.20	**

Abbreviation: HCC, hepatocellular carcinoma.

* *p* < .05

** *p* < .01.

### 3.7 Constructing and evaluating prognostic model

**Figure 8:**
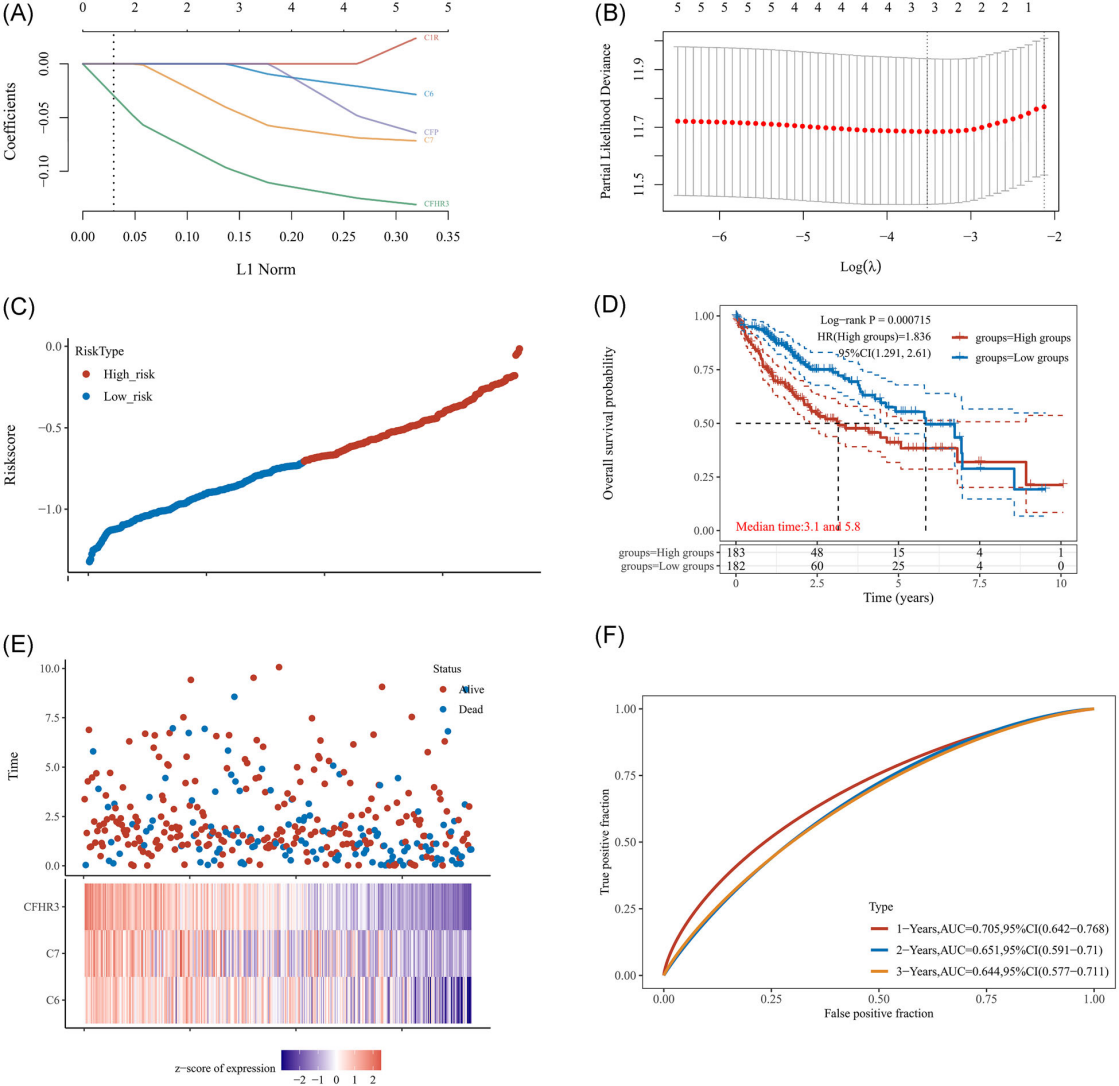
Prognostic Model based on the expression of C1R, C6, C7, CFP, and CFHR3 in HCC patients. (A) Coefficients of selected genes. (B) Partial likelihood deviance. (C) Risk Score for all HCC patients in TCGA. (D) K–M survival curve for OS using the prognostic model. (E) Heatmap of the expression profiles of the prognostic genes in low‐ and high‐risk groups. (F) Time‐dependent ROC analysis of the model. HCC, hepatocellular carcinoma; OS, overall survival; TCGA, The Cancer Genome Atlas

As we thought the gene expressions of C1R, C6, C7, CFP, and CFHR3 had prognostic value in HCC, we used LASSO Cox regression to build the model. Based on OS information of patients with HCC, we generated the prognostic model: Riskscore = (−0.0053)*C6+(−0.0498)*C7+(−0.1045)*CFHR3, with a lambda.min = 0.0296 (Figure 8). As higher scores related to poorer OS in this model (Figure 8C).

Next, we verified the model by K–M survival analysis and time‐dependent ROC analysis. We drew the K–M survival curve using the medium cutoff value of our Risk Score. As shown in Figure 8D, the medium survive months in the higher score group was significantly shorter than that in the lower score group (3.1 vs. 5.8 months, *p* < .01). Also, the time‐dependent ROC analysis showed that the 1‐year AUC of this model was 0.705 (95% CI = 0.642–0.768; Figure 8F).

## 4 DISCUSSION

Recently, several studies have revealed the imbalance of the complement system may affect the human immune system.^35^, ^36^ Its abnormal activation might be the cause of many human diseases, including different kinds of cancers.^37^ As there was no prior study focusing on evaluating the relationship between the complement system and HCC, we examined the mRNA expressions of 47 complement genes in patients with HCC through the public database to draw a general landscape of the complement system in HCC in this study.

We found that there were six complement genes, including C1R, C6, C7, C9, CFP, and CFHR3, whose mRNA expressions were significantly lower in HCC samples than in their normal tissue counterparts according to three data sets—TIMER2, GEPIA, and GEO with a fold change >2 or <0.5 and *p* < .05. Bao et al.^38^ reported that many complement genes such as C3, C4R, C5aR1 were overexpressed in gastric and colon cancer compared with normal tissues. Mangogna et al.^39^ reported that C1q was overexpressed in breast cancer and kidney cancer, while in lung cancer, a lower level of C1q expression was found. We suspected the low mRNA expressions of complement genes in HCC might result from the fact that liver is an organ with predominant innate immunity full of complement proteins.^40^ As HCC cells are immature, they cannot synthesize complement proteins as normal hepatocytes. Morris et al.^41^ examined the complement biosynthesis function of a well‐differentiated HCC cell line—HepG2 and found that HepG2 synthesized and secreted functional complement proteins C1r, C1s, C2, C3, C4, C5, factor B, C1 inhibitor, C3b inactivator, a small amount of C6, and trace amounts of C8; but failed to produce detectable C1q, C7, or C9,^42^ which supported our hypothesis. Nwosu's study showed that poorly‐differentiated HCC cell lines are phenotypically more “cancer‐like” and possess more tumor molecular portraits than well‐differentiated HCC cell lines,^43^ which might lead poorly‐differentiated HCC cells to preserve less normal liver cell functions than well‐differentiated HCC cells, for example, less complement biosynthesis. However, further studies are still needed to elucidate the relationship between HCC differentiation and complement biosynthesis function.

In this study, only six complement genes were found downregulated in HCC and only five of them (C1R, C6, C7, CFP, and CFHR3) were considered to have prognostic value. We used KEGG analysis to further speculate the biological function of C1R, C6, C7, CFP, and CFHR3 in HCC. We found that the lower mRNA expressions of C1R, C6, C7, CFP, and CFHR3 were correlated with pathways like Fanconi anemia pathway, cell cycle, MicroRNAs in cancers, and so on. These pathways were reported to promote proliferation, migration, and invasion in HCC.^44^, ^45^ According to these results, we suspected these five complement genes might participate in the regulation of HCC through these pathways.

C1R, a complement protein, combined with C1q and C1S to activate the classical pathway of the complement system. Our study found its prognostic value in patients with HCC. A previous study had shown the serum concentration of C1R had prognostic value in non‐small cell lung cancer.^46^ It is reported that the knockdown of C1r promotes apoptosis of cutaneous squamous cell carcinoma cells.^47^ But its biological function in HCC is not very clear. So future study focusing on the biological mechanism of C1R in HCC is needed.

C6 is a complement protein composing the membrane attack complex (MAC). Our study showed its prognostic value in HCC patients, which was consistent with other studies like Mu et al.^42^ However, to our knowledge, little research has been done on the biological function of C6 in HCC.^48^ It was reported that cell line HepG2—an HCC cell line, secreted only a small amount of C6, much lower than other complement proteins,^41^ indicating it might interfere with the biological functions of HCC.

C7 is also a complement protein composing MAC. Our study showed the prognostic value of mRNA expression of C7 in HCC. Lisha et al.^49^ showed that C7 was not only a prognostic marker but also suppressed tumor growth in non‐small cell lung cancer. Chen et al.^50^ showed C7 was a prognostic marker in prostate cancer. Also, Zhao et al.^51^ showed C7 peptide inhibited HCC metastasis by targeting the HGF/c‐Met signaling pathway, which indicated its antitumor function in HCC.

CFP, also known as properdin—activating the alternative pathway of the complement system by combining C3bBb, was also reported to have prognostic value in HCC, lung adenocarcinoma, and pancreatic adenocarcinoma.^52^ The higher mRNA expression of CFP was associated with better OS in these cancers, which was also consistent with our results. Block et al.^53^ reported that CFP could suppress breast cancer cell growth by upregulating the transcription factor DDIT3 in vitro and in vivo. Also, Al‐Rayahi et al.^54^ showed that properdin (CFP) insufficiency promotes greater M2 skewing of macrophages, causing a tumor environment that helped the tumor evade the immune response. Our result also showed its mRNA expression had correlations with tumor immune cells, including M2 macrophages, which might indicate the possible mechanism of CFP in regulating tumor immune cells in hepatocellular carcinoma.

CFHR3, a negative regulator of the complement system, was reported to have prognostic value in patients with HCC according to our study. Other studies, like Liu et al.^55^ also reported the prognostic value of CFHR3 in HCC. Previous studies have reported it could inhibit proliferation and introduce apoptosis in HCC via inhibiting the P13K/Akt pathway.^56^ As our result showed higher mRNA expression of CFHR3 was associated with better outcomes in patients with HCC, it might be a therapy target for HCC.

The mRNA expressions of MBL2 and C8A were significantly downregulated in HCC samples than in normal liver tissues in Gepia database with a fold change >2 or <0.5 and *p* < .05, but not according to GSE25097 (although *p* < .05, fold change ranged from 0.5 to 2). Similarly, the mRNA expression of MASP1 was significantly downregulated in HCC samples than in normal liver tissues in GSE25097 with a fold change >2 or <0.5 and *p* < .05, but not according to Gepia database (although *p* < .05, fold change ranged from 0.5 to 2). So, we did not consider their mRNA expressions were significantly different between HCC samples and normal liver tissues in this study, but we believed further research are needed to confirm the mRNA expressions of MBL2, C8A, and MASP1 in HCC as they might have an important biological function in HCC. Transcription levels of other complement genes, like CD46, CD59, and C1QBP, were found up‐ or downregulated in HCC samples only in one data set—TIMER2, without a fold change >0.5 and <2. So, we did not believe the expression of these genes was significantly altered in HCC.

Here, we reported that five complement genes showed prognostic values in patients with HCC, including C1R, C6, C7, CFP, and CFHR3. As shown in our result, the mRNA expressions of these five genes were significantly lower in HCC samples than in normal controlled samples. Moreover, lower expression of C1R, C6, C7, CFP, and CFHR3 was associated with poorer OS, DFS, and PFS in HCC patients. Also, we found that the mRNA expression of C1R, C6, C7, and CFHR3 was associated with tumor grade, while the mRNA expression of C1R, C6, and CFHR3 was associated with the cancer stage. So, we thought C1R, C6, C7, and CFHR3 could be prognostic biomarkers for patients with HCC. Although CFP was not associated with tumor grade or cancer stage, it was associated with the mRNA expressions of many immune marker genes in HCC, including CD8+ T cells (CD8A, CD8B), CD4+ T cells (CD3E, CD2), B cells (CD79A), M2 macrophages (CD163), neutrophils (CCR7), DCs (CD1C, HLA‐DPA1, HLA‐DPB1, HLA‐DQB1, HLA‐DRA), Th1 cells (IFNG, TBX21), Th2 cells (GATA3) and T cell exhaustion (GZMB, LAG3, PDCD1), and so on. Thus, we thought CFP could also be a biomarker for HCC patients. As higher CFP expression was associated with better OS in HCC patients, we speculated it might be associated with the immune system regulating HCC and might be a new target or biomarker for immune therapy. Furthermore, we developed a prognostic model for HCC patients using these complement genes.

Moreover, mRNA expressions of many complement genes were not altered or unclear according to our results. However, these genes were also found associated with OS, DFS, and PFS in HCC patients, like C2, C3, C5, and so on. Thus, we further examined these genes and found that the mRNA expressions of C3, C5, CFB, and CLU were correlated with cancer stage and prognosis in patients with HCC. In addition, the mRNA expressions of C1S, C8B, MBL2, CFI, and C4BPA were correlated with tumor grade and prognosis in HCC patients. Other studies, like Wang et al.^57^ showed that CLU had prognostic value in HCC. Chen et al.^58^ showed that C3 also had prognostic value in HCC. Thus, we considered these complement genes might also serve as prognostic biomarkers in clinic after further validation.

Apart from complement genes mentioned above, the mRNA expressions of C2, CFH, CFHR1, CFHR2, CFHR4, SERPING1, MASP2, MASP1, C4BPB, VTN, CPN1, and CR2 were associated with OS, DFS, and PFS in patients with HCC. However, their mRNA expressions were neither altered in HCC, nor associated with cancer stage or tumor grade in HCC. As result, we did not consider these genes had prognostic value in HCC.

Generally, it is believed that the activation of the complement system is associated with tumorigenesis, progression, and so on, by causing inflammation in the tumor microenvironment (TME).^37^ Studies have shown the connection between C3/C3aR, C5/C5aR, and the development of HCC.^59^ Other study has shown that complement suppresses various antitumor immune cells through C3aR and C5aR. Markiewski et al.^60^ first showed that C5a/C5aR1 interaction promotes the migration of Myeloid‐derived suppressor cells (MDSCs) into tumors and enhances the suppressive capacity of tumor‐associated MDSCs. Janelle et al.^61^ also revealed that CD4+ and CD8+ TILs are enhanced in the complement signaling deficient mice. According to our study, we did find that the mRNA expressions of C5AR1 and C3AR1 were positively correlated with almost all kinds of tumor immune cells, including CD8+ T cells, CD4+ T cells, B cells, macrophages, monocytes, and DCs (Table 3). Although the OS of HCC patients was not associated with the mRNA expression of C3aR and C5aR, the median survival months in C3aR and C5aR high expression group were shorter than that of the low expression group in our study (Table 1). Further study would be needed to confirm its function in patients with HCC.

There were some limitations in our study. First, all the data was retrieved from online public databases (TCGA and GEO), further studies with larger sample sizes are required to validate and to explore the application of the five complement members in the prognosis and treatment of HCC. Second, we did not explore the possible mechanisms of these five complement genes in HCC via basic experiments. Finally, 27 complement genes other than C1R, C6, C7, CFP, and CFHR3 were also associated with better OS and might have an important biological function in HCC. We were intended to confirm their role in HCC and investigate the mechanism between these genes and HCC in further study.

## 5 CONCLUSION

In summary, we assessed the gene expressions of the complement system in HCC. We have found C1R, C6, C7, CFP, and CFHR3 have prognostic value as they were not only significantly downregulated in HCC but also correlated with better OS, DFS, and PFS in HCC patients. Also, C1R, C6, C7, and CFHR3 presented correlations with tumor grades and cancer stage in HCC patients, while CFP presented correlations with the immune markers of tumor immune cells in HCC. Furthermore, we constructed a prognostic model based on our results: Riskscore = (−0.0053)*C6+(−0.0498)*C7+(−0.1045)*CFHR3.

## ETHICS STATEMENT

The data of the patients in this study were obtained from the public database data sets.

## AUTHOR CONTRIBUTIONS

Xinye Qian and Zhoujing Yang conceived and designed the study; Lu Gao and Yipiao Liu downloaded and organized The Cancer Genome Atlas data; Xinye Qian and Zhoujing Yang performed data analysis and wrote the paper; Jun Yan critically revised the article for essential intellectual content and administrative support. All authors read and approved the final manuscript.

## Supporting information

Supporting information.

## Data Availability

The data sets used and/or analyzed during the current study are available from the corresponding author on reasonable request.

## References

[1] Sung H , Ferlay J , Siegel RL , et al. Global Cancer Statistics 2020: GLOBOCAN estimates of incidence and mortality worldwide for 36 cancers in 185 countries. CA Cancer J Clin. 2021;71:209‐249. 33538338 10.3322/caac.21660

[2] Forner A , Reig M , Bruix J . Hepatocellular carcinoma. Lancet. 2018;391:1301‐1314. 29307467 10.1016/S0140-6736(18)30010-2

[3] van den Bulk J , Verdegaal EM , de Miranda NF . Cancer immunotherapy: broadening the scope of targetable tumours. Open Biol. 2018;8. 10.1098/rsob.180037 PMC6030119 29875199

[4] Vedham V , Verma M . Cancer‐associated infectious agents and epigenetic regulation. Methods Mol Biol. 2015;1238:333‐354. 25421669 10.1007/978-1-4939-1804-1_18

[5] Ma L , Chua MS , Andrisani O , So S . Epigenetics in hepatocellular carcinoma: an update and future therapy perspectives. World J Gastroenterol. 2014;20:333‐345. 24574704 10.3748/wjg.v20.i2.333 PMC3923010

[6] Finn RS , Qin S , Ikeda M , et al. Atezolizumab plus bevacizumab in unresectable hepatocellular carcinoma. N Engl J Med. 2020;382:1894‐1905. 32402160 10.1056/NEJMoa1915745

[7] Sarma JV , Ward PA . The complement system. Cell Tissue Res. 2011;343:227‐235. 20838815 10.1007/s00441-010-1034-0 PMC3097465

[8] Huber‐Lang M , Younkin EM , Sarma JV , et al. Generation of C5a by phagocytic cells. Am J Pathol. 2002;161:1849‐1859. 12414531 10.1016/S0002-9440(10)64461-6 PMC1850785

[9] Huber‐Lang M , Sarma JV , Zetoune FS , et al. Generation of C5a in the absence of C3: a new complement activation pathway. Nat Med. 2006;12:682‐687. 16715088 10.1038/nm1419

[10] Afshar‐Kharghan V . The role of the complement system in cancer. J Clin Invest. 2017;127:780‐789. 28248200 10.1172/JCI90962 PMC5330758

[11] Taylor RP , Lindorfer MA . Cytotoxic mechanisms of immunotherapy: harnessing complement in the action of anti‐tumor monoclonal antibodies. Semin Immunol. 2016;28:309‐316. 27009480 10.1016/j.smim.2016.03.003

[12] Di Gaetano N , Cittera E , Nota R , et al. Complement activation determines the therapeutic activity of rituximab in vivo. J Immunol. 2003;171:1581‐1587. 12874252 10.4049/jimmunol.171.3.1581

[13] Coussens LM , Werb Z . Inflammation and cancer. Nature. 2002;420:860‐867. 12490959 10.1038/nature01322 PMC2803035

[14] Zhang R , Liu Q , Li T , Liao Q , Zhao Y . Role of the complement system in the tumor microenvironment. Cancer Cell Int. 2019;19:300. 31787848 10.1186/s12935-019-1027-3 PMC6858723

[15] Cho MS , Vasquez HG , Rupaimoole R , et al. Autocrine effects of tumor‐derived complement. Cell Rep. 2014;6:1085‐1095. 24613353 10.1016/j.celrep.2014.02.014 PMC4084868

[16] Habermann JK , Roblick UJ , Luke BT , et al. Increased serum levels of complement C3a anaphylatoxin indicate the presence of colorectal tumors. Gastroenterology. 2006;131:1020‐1029. 17030172 10.1053/j.gastro.2006.07.011 PMC2532535

[17] Corrales L , Ajona D , Rafail S , et al. Anaphylatoxin C5a creates a favorable microenvironment for lung cancer progression. J Immunol. 2012;189:4674‐4683. 23028051 10.4049/jimmunol.1201654 PMC3478398

[18] Kusmartsev S , Nefedova Y , Yoder D , Gabrilovich DI . Antigen‐specific inhibition of CD8+ T cell response by immature myeloid cells in cancer is mediated by reactive oxygen species. J Immunol. 2004;172:989‐999. 14707072 10.4049/jimmunol.172.2.989

[19] Togashi Y , Shitara K , Nishikawa H . Regulatory T cells in cancer immunosuppression – implications for anticancer therapy. Nat Rev Clin Oncol. 2019;16:356‐371. 30705439 10.1038/s41571-019-0175-7

[20] Li Y , Zhu S , Xue M , et al. Aristolochic acid I promotes the invasion and migration of hepatocellular carcinoma cells by activating the C3a/C3aR complement system. Toxicol Lett. 2020 10.1016/j.toxlet.2020.08.014 32898628

[21] Chen B , Zhou W , Tang C , et al. Down‐regulation of C3aR/C5aR inhibits cell proliferation and EMT in hepatocellular carcinoma. Technol Cancer Res Treat. 2020;19:1533033820970668. 33176600 10.1177/1533033820970668 PMC7672723

[22] Laskowski J , Renner B , Pickering MC , et al. Complement factor H‐deficient mice develop spontaneous hepatic tumors. J Clin Invest. 2020;130:4039‐4054. 32369457 10.1172/JCI135105 PMC7410061

[23] Malik A , Thanekar U , Amarachintha S , et al. "Complimenting the complement": mechanistic insights and opportunities for therapeutics in hepatocellular carcinoma. Front Oncol. 2020;10:627701. 33718121 10.3389/fonc.2020.627701 PMC7943925

[24] Tomczak K , Czerwińska P , Wiznerowicz M . The Cancer Genome Atlas (TCGA): an immeasurable source of knowledge. Contemp Oncol. 2015;19:A68‐A77. 10.5114/wo.2014.47136 PMC4322527 25691825

[25] Lee SY , Song KH , Koo I , Lee KH , Suh KS , Kim BY . Comparison of pathways associated with hepatitis B‐ and C‐infected hepatocellular carcinoma using pathway‐based class discrimination method. Genomics. 2012;99:347‐354. 22564472 10.1016/j.ygeno.2012.04.004

[26] Li T , Fu J , Zeng Z , et al. TIMER2.0 for analysis of tumor‐infiltrating immune cells. Nucleic Acids Res. 2020;48:W509‐W514. 32442275 10.1093/nar/gkaa407 PMC7319575

[27] Tang Z , Li C , Kang B , Gao G , Li C , Zhang Z . GEPIA: a web server for cancer and normal gene expression profiling and interactive analyses. Nucleic Acids Res. 2017;45:W98‐W102. 28407145 10.1093/nar/gkx247 PMC5570223

[28] Chandrashekar DS , Bashel B , Balasubramanya SAH , et al. UALCAN: a portal for facilitating tumor subgroup gene expression and survival analyses. Neoplasia. 2017;19:649‐658. 28732212 10.1016/j.neo.2017.05.002 PMC5516091

[29] Győrffy B , Surowiak P , Budczies J , Lánczky A . Online survival analysis software to assess the prognostic value of biomarkers using transcriptomic data in non‐small‐cell lung cancer. PLOS One. 2013;8:e82241. 24367507 10.1371/journal.pone.0082241 PMC3867325

[30] Nagy Á , Lánczky A , Menyhárt O , Győrffy B . Validation of miRNA prognostic power in hepatocellular carcinoma using expression data of independent datasets. Sci Rep. 2018;8:9227. 29907753 10.1038/s41598-018-27521-y PMC6003936

[31] Gao J , Aksoy BA , Dogrusoz U , et al. Integrative analysis of complex cancer genomics and clinical profiles using the cBioPortal. Sci Signal. 2013;6:pl1. 23550210 10.1126/scisignal.2004088 PMC4160307

[32] Szklarczyk D , Gable AL , Lyon D , et al. STRING v11: protein‐protein association networks with increased coverage, supporting functional discovery in genome‐wide experimental datasets. Nucleic Acids Res. 2019;47:D607‐D613. 30476243 10.1093/nar/gky1131 PMC6323986

[33] Vasaikar SV , Straub P , Wang J , Zhang B . LinkedOmics: analyzing multi‐omics data within and across 32 cancer types. Nucleic Acids Res. 2018;46:D956‐D963. 29136207 10.1093/nar/gkx1090 PMC5753188

[34] Zhou Y , Zhou B , Pache L , et al. Metascape provides a biologist‐oriented resource for the analysis of systems‐level datasets. Nat Commun. 2019;10:1523. 30944313 10.1038/s41467-019-09234-6 PMC6447622

[35] Carroll MC , Isenman DE . Regulation of humoral immunity by complement. Immunity. 2012;37:199‐207. 22921118 10.1016/j.immuni.2012.08.002 PMC5784422

[36] West EE , Kolev M , Kemper C . Complement and the regulation of T cell responses. Annu Rev Immunol. 2018;36:309‐338. 29677470 10.1146/annurev-immunol-042617-053245 PMC7478175

[37] Reis ES , Mastellos DC , Ricklin D , Mantovani A , Lambris JD . Complement in cancer: untangling an intricate relationship. Nat Rev Immunol. 2018;18:5‐18. 28920587 10.1038/nri.2017.97 PMC5816344

[38] Bao D , Zhang C , Li L , et al. Integrative analysis of complement system to prognosis and immune infiltrating in colon cancer and gastric cancer. Front Oncol. 2020;10:553297. 33614473 10.3389/fonc.2020.553297 PMC7886994

[39] Mangogna A , Agostinis C , Bonazza D , et al. Is the complement protein C1q a pro‐ or anti‐tumorigenic factor? Bioinformatics analysis involving human carcinomas. Front Immunol. 2019;10:865. 31130944 10.3389/fimmu.2019.00865 PMC6509152

[40] Gao B , Jeong WI , Tian Z . Liver: an organ with predominant innate immunity. Hepatology. 2008;47:729‐736. 18167066 10.1002/hep.22034

[41] Morris KM , Aden DP , Knowles BB , Colten HR . Complement biosynthesis by the human hepatoma‐derived cell line HepG2. J Clin Invest. 1982;70:906‐913. 6288774 10.1172/JCI110687 PMC370299

[42] Mu D , Qin F , Li B , Zhou Q . Identification of the sixth complement component as potential key genes in hepatocellular carcinoma via bioinformatics analysis. BioMed Res Int. 2020;2020:7042124. 33083480 10.1155/2020/7042124 PMC7556077

[43] Nwosu ZC , Battello N , Rothley M , et al. Liver cancer cell lines distinctly mimic the metabolic gene expression pattern of the corresponding human tumours. J Exp Clin Cancer Res. 2018;37:211. 30176945 10.1186/s13046-018-0872-6 PMC6122702

[44] Ferroudj S , Yildiz G , Bouras M , Iscan E , Ekin U , Ozturk M . Role of Fanconi anemia/BRCA pathway genes in hepatocellular carcinoma chemoresistance. Hepatol Res. 2016;46:1264‐1274. 26885668 10.1111/hepr.12675

[45] Li D , Zhang J , Li J . Role of miRNA sponges in hepatocellular carcinoma. Clin Chim Acta. 2020;500:10‐19. 31604064 10.1016/j.cca.2019.09.013

[46] Gürz S , Çelik B , Menteşe A , Us Altay D . Diagnostic value of signal peptide‐Complement C1r/C1s, Uegf, and Bmp1‐epidermal growth factor domain‐containing protein 1 on serum and tissue samples in non‐small cell lung cancer. Turk Gogus Kalp Damar Cerrahisi Derg. 2018;26:246‐253. 32082741 10.5606/tgkdc.dergisi.2018.14600 PMC7024133

[47] Riihilä P , Viiklepp K , Nissinen L , et al. Tumour‐cell‐derived complement components C1r and C1s promote growth of cutaneous squamous cell carcinoma. Br J Dermatol. 2020;182:658‐670. 31049937 10.1111/bjd.18095 PMC7065064

[48] Cook MB , Dawsey SM , Freedman ND , et al. Sex disparities in cancer incidence by period and age. Cancer Epidemiol Biomarkers Prev. 2009;18:1174‐1182. 19293308 10.1158/1055-9965.EPI-08-1118 PMC2793271

[49] Ying L , Zhang F , Pan X , et al. Complement component 7 (C7), a potential tumor suppressor, is correlated with tumor progression and prognosis. Oncotarget. 2016;7:86536‐86546. 27852032 10.18632/oncotarget.13294 PMC5349933

[50] Chen Z , Yan X , Du GW , et al. Complement C7 (C7), a potential tumor suppressor, is an immune‐related prognostic biomarker in prostate cancer (PC). Front Oncol. 2020;10:1532. 32984006 10.3389/fonc.2020.01532 PMC7477933

[51] Zhao M , Wang Y , Liu Y , et al. C7 peptide inhibits hepatocellular carcinoma metastasis by targeting the HGF/c‐Met signaling pathway. Cancer Biol Ther. 2019;20:1430‐1442. 31441380 10.1080/15384047.2019.1647051 PMC6804814

[52] Mangogna A , Varghese PM , Agostinis C , et al. Prognostic value of complement properdin in cancer. Front Immunol. 2020;11:614980. 33542722 10.3389/fimmu.2020.614980 PMC7851055

[53] Block I , Müller C , Sdogati D , et al. CFP suppresses breast cancer cell growth by TES‐mediated upregulation of the transcription factor DDIT3. Oncogene. 2019;38:4560‐4573. 30755730 10.1038/s41388-019-0739-0

[54] Al‐Rayahi IA , Browning MJ , Stover C . Tumour cell conditioned medium reveals greater M2 skewing of macrophages in the absence of properdin. Immun Inflamm Dis. 2017;5:68‐77. 28250926 10.1002/iid3.142 PMC5322164

[55] Liu J , Li W , Zhao H . CFHR3 is a potential novel biomarker for hepatocellular carcinoma. J Cell Biochem. 2020;121:2970‐2980. 31709629 10.1002/jcb.29551

[56] Liu H , Zhang L , Wang P . Complement factor H‑related 3 overexpression affects hepatocellular carcinoma proliferation and apoptosis. Mol Med Rep. 2019;20:2694‐2702. 31524260 10.3892/mmr.2019.10514 PMC6691229

[57] Wang X , Liu Y , Qin Q , Zheng T . Clusterin role in hepatocellular carcinoma patients treated with oxaliplatin. Biosci Rep. 2020;40. 10.1042/BSR20200071 PMC7033306 32039450

[58] Chen Q , Li F , Gao Y , Xu G , Liang L , Xu J . Identification of energy metabolism genes for the prediction of survival in hepatocellular carcinoma. Front Oncol. 2020;10:1210. 32903581 10.3389/fonc.2020.01210 PMC7438573

[59] Bröker K , Terzenbach R , Bentzien F , Lüth S , Dammermann W . Complement factors C3a and C5a mimick a proinflammatory microenvironment and increase HBV IGRA sensitivity. J Transl Med. 2019;17:6. 30602374 10.1186/s12967-018-1752-8 PMC6317231

[60] Markiewski MM , DeAngelis RA , Benencia F , et al. Modulation of the antitumor immune response by complement. Nat Immunol. 2008;9:1225‐1235. 18820683 10.1038/ni.1655 PMC2678913

[61] Janelle V , Langlois MP , Tarrab E , Lapierre P , Poliquin L , Lamarre A . Transient complement inhibition promotes a tumor‐specific immune response through the implication of natural killer cells. Cancer Immunol Res. 2014;2:200‐206. 24778316 10.1158/2326-6066.CIR-13-0173

