# Supplementary material for: The role of complement in the clinical course of hepatocellular carcinoma
Source: Immun Inflamm Dis. 2021 Nov 23;10(3):e569. doi: 10.1002/iid3.569 (PMC8926509; doi:10.1002/iid3.569)
Supplement: Supplementary file 1 — Supporting information. [file IID3-10-e569-s001.docx]

**Supplement Table 3**

**Supplementary table 1. 50 most co-expressed genes of C1R, C6, C7, CFP and CFHR3.**

| gene name | positively correlated gene | spearsman coefficient | P value | negetively correlated gene | spearsman coefficient | P value |
| --- | --- | --- | --- | --- | --- | --- |
| C1R | C1S | 0.862778432 | 2.23E-108 | ILF3 | -0.580098229 | 7.53E-34 |
|  | SERPING1 | 0.708788466 | 2.32E-56 | TCF3 | -0.578780704 | 1.14E-33 |
|  | C1RL | 0.703260697 | 3.82E-55 | BEND3 | -0.549734504 | 6.76E-30 |
|  | HP | 0.656417978 | 7.31E-46 | NFYA | -0.542407786 | 5.30E-29 |
|  | SAA1 | 0.648349964 | 1.99E-44 | ABCC10 | -0.537485164 | 2.06E-28 |
|  | DHODH | 0.644687192 | 8.60E-44 | COPS7B | -0.530897593 | 1.22E-27 |
|  | SAA2 | 0.624068344 | 2.30E-40 | ZBTB12 | -0.523737658 | 8.08E-27 |
|  | TNFRSF1A | 0.595910739 | 4.46E-36 | TARBP1 | -0.517965591 | 3.59E-26 |
|  | CFB | 0.590209844 | 2.93E-35 | DVL3 | -0.513464361 | 1.13E-25 |
|  | C4A | 0.587984059 | 6.05E-35 | LZTS2 | -0.5087969 | 3.63E-25 |
|  | C3P1 | 0.586293445 | 1.04E-34 | WHSC2 | -0.507494871 | 5.01E-25 |
|  | NNMT | 0.583034033 | 2.97E-34 | SPATS2 | -0.506636086 | 6.19E-25 |
|  | C6 | 0.58272338 | 3.28E-34 | CCDC97 | -0.505367661 | 8.46E-25 |
|  | ITIH4 | 0.567880995 | 3.29E-32 | CPSF6 | -0.505057827 | 9.13E-25 |
|  | C9 | 0.560727518 | 2.79E-31 | PIK3R2 | -0.504932693 | 9.42E-25 |
|  | ECHDC2 | 0.557425594 | 7.36E-31 | SMARCC1 | -0.502622979 | 1.66E-24 |
|  | CFI | 0.555019274 | 1.48E-30 | SMPD4 | -0.501315473 | 2.27E-24 |
|  | MFSD2A | 0.552268017 | 3.28E-30 | ATXN7L3 | -0.500335196 | 2.88E-24 |
|  | CFHR3 | 0.552166267 | 3.37E-30 | CENPF | -0.49826542 | 4.75E-24 |
|  | ANXA10 | 0.550805239 | 4.98E-30 | ZNF74 | -0.493969682 | 1.32E-23 |
|  | CP | 0.549766652 | 6.70E-30 | DNMT3B | -0.493953497 | 1.33E-23 |
|  | ACAT1 | 0.549390617 | 7.46E-30 | MEX3A | -0.49214591 | 2.03E-23 |
|  | BLVRB | 0.549195926 | 7.88E-30 | C2orf29 | -0.489914857 | 3.42E-23 |
|  | RARRES3 | 0.548352523 | 1.00E-29 | PLAGL2 | -0.489062617 | 4.18E-23 |
|  | SOD2 | 0.54787504 | 1.15E-29 | GTF2IRD1 | -0.487690258 | 5.74E-23 |
|  | PINK1 | 0.546689383 | 1.60E-29 | UBAP2L | -0.48529526 | 9.99E-23 |
|  | CD14 | 0.546125934 | 1.88E-29 | CBX1 | -0.484816769 | 1.11E-22 |
|  | CFHR4 | 0.540064406 | 1.01E-28 | LOC642852 | -0.48280295 | 1.77E-22 |
|  | DHRS3 | 0.539410342 | 1.21E-28 | C11orf93 | -0.482034904 | 2.10E-22 |
|  | LOC100131726 | 0.535289598 | 3.74E-28 | UBAP2 | -0.481386583 | 2.44E-22 |
|  | HPR | 0.531310386 | 1.09E-27 | CCDC93 | -0.480689739 | 2.86E-22 |
|  | IL13RA1 | 0.530445974 | 1.38E-27 | UBA2 | -0.47954312 | 3.70E-22 |
|  | CTSO | 0.530216229 | 1.46E-27 | BAT2 | -0.478624239 | 4.55E-22 |
|  | APOL1 | 0.529668772 | 1.69E-27 | BUB1B | -0.478475264 | 4.71E-22 |
|  | CXCL2 | 0.529337458 | 1.85E-27 | ZBED4 | -0.47717052 | 6.31E-22 |
|  | LDHD | 0.528380681 | 2.38E-27 | ANKRD52 | -0.477009731 | 6.54E-22 |
|  | RDH16 | 0.526295521 | 4.14E-27 | C12orf41 | -0.476965248 | 6.60E-22 |
|  | SLC27A5 | 0.522868486 | 1.01E-26 | SMARCD1 | -0.476960321 | 6.61E-22 |
|  | GSTZ1 | 0.521627988 | 1.40E-26 | TET3 | -0.475458222 | 9.24E-22 |
|  | ALPL | 0.519190297 | 2.63E-26 | PBX2 | -0.475236085 | 9.71E-22 |
|  | MGST1 | 0.517531164 | 4.02E-26 | SOX12 | -0.47403162 | 1.27E-21 |
|  | CCBP2 | 0.516080875 | 5.81E-26 | USP39 | -0.473317474 | 1.49E-21 |
|  | SLC46A3 | 0.514288442 | 9.16E-26 | TRIM71 | -0.47258593 | 1.75E-21 |
|  | TAPBPL | 0.513149997 | 1.22E-25 | DNMT3A | -0.471646922 | 2.15E-21 |
|  | IGFBP4 | 0.51259264 | 1.41E-25 | LMNB2 | -0.471621318 | 2.16E-21 |
|  | ADH1B | 0.511894488 | 1.67E-25 | PIP4K2B | -0.471213593 | 2.36E-21 |
|  | TAT | 0.511723221 | 1.75E-25 | USP21 | -0.470399489 | 2.82E-21 |
|  | ORM1 | 0.511700321 | 1.76E-25 | ANLN | -0.469992086 | 3.09E-21 |
|  | HAAO | 0.511116452 | 2.04E-25 | KIF20B | -0.469972924 | 3.10E-21 |
|  | CFHR5 | 0.510532577 | 2.36E-25 | LIN9 | -0.469079005 | 3.77E-21 |
| C6 | CFHR4 | 0.686633083 | 1.20E-51 | SPATS2 | -0.59351197 | 9.89E-36 |
|  | TAT | 0.66137327 | 9.14E-47 | SMARCD1 | -0.582137774 | 3.95E-34 |
|  | GYS2 | 0.647653049 | 2.63E-44 | TPD52L2 | -0.575637301 | 3.05E-33 |
|  | SERPING1 | 0.644679842 | 8.63E-44 | LMNB2 | -0.569123862 | 2.25E-32 |
|  | KLKB1 | 0.636969458 | 1.77E-42 | RCC2 | -0.56764374 | 3.53E-32 |
|  | HP | 0.634109177 | 5.32E-42 | TMEM201 | -0.562373307 | 1.71E-31 |
|  | ANXA10 | 0.630837465 | 1.84E-41 | MAPRE1 | -0.558360029 | 5.60E-31 |
|  | GLYATL1 | 0.630218623 | 2.33E-41 | MYBL2 | -0.555786885 | 1.19E-30 |
|  | SLC10A1 | 0.629381758 | 3.19E-41 | NCAPD2 | -0.554656718 | 1.65E-30 |
|  | MOSC2 | 0.62895164 | 3.75E-41 | SMPD4 | -0.553102615 | 2.58E-30 |
|  | F9 | 0.627106243 | 7.47E-41 | C11orf84 | -0.549951096 | 6.36E-30 |
|  | LDHD | 0.627046461 | 7.64E-41 | CEP55 | -0.549402929 | 7.43E-30 |
|  | ITIH4 | 0.626129356 | 1.07E-40 | AURKB | -0.547429662 | 1.30E-29 |
|  | CFHR3 | 0.620302988 | 9.14E-40 | CDCA4 | -0.54608765 | 1.90E-29 |
|  | HPX | 0.618231432 | 1.94E-39 | E2F6 | -0.538201823 | 1.69E-28 |
|  | HSD17B6 | 0.615813883 | 4.61E-39 | ANKLE2 | -0.537743712 | 1.92E-28 |
|  | C1S | 0.613533732 | 1.04E-38 | PLK1 | -0.537300956 | 2.16E-28 |
|  | ACSM2A | 0.607694118 | 8.08E-38 | UBE2C | -0.536883107 | 2.43E-28 |
|  | C3P1 | 0.606156959 | 1.38E-37 | BEND3 | -0.536768354 | 2.50E-28 |
|  | GLYAT | 0.605910383 | 1.50E-37 | ILF3 | -0.535893748 | 3.17E-28 |
|  | CFH | 0.605714954 | 1.60E-37 | TCF3 | -0.534507486 | 4.62E-28 |
|  | ECHDC2 | 0.604835752 | 2.17E-37 | CDCA7 | -0.533099924 | 6.76E-28 |
|  | C8A | 0.604423741 | 2.50E-37 | LASS5 | -0.532562665 | 7.81E-28 |
|  | ALDOB | 0.603300136 | 3.68E-37 | KIF2C | -0.531578771 | 1.02E-27 |
|  | HAO1 | 0.601770541 | 6.20E-37 | GTSE1 | -0.531086648 | 1.16E-27 |
|  | CYP4F2 | 0.600044118 | 1.11E-36 | C12orf32 | -0.530813763 | 1.25E-27 |
|  | TTC36 | 0.598116963 | 2.13E-36 | RACGAP1 | -0.530517855 | 1.35E-27 |
|  | HRSP12 | 0.597847285 | 2.33E-36 | TRIP13 | -0.530120191 | 1.50E-27 |
|  | ACSM2B | 0.59671948 | 3.41E-36 | BUB1B | -0.52838425 | 2.38E-27 |
|  | GHR | 0.595370535 | 5.34E-36 | COPS7B | -0.525665459 | 4.88E-27 |
|  | C8B | 0.595200146 | 5.65E-36 | SMYD5 | -0.523576598 | 8.43E-27 |
|  | SLC27A5 | 0.594745525 | 6.57E-36 | CDCA3 | -0.523170559 | 9.37E-27 |
|  | CFB | 0.59409293 | 8.16E-36 | HJURP | -0.521235272 | 1.55E-26 |
|  | BDH1 | 0.591848951 | 1.71E-35 | C15orf42 | -0.52100919 | 1.64E-26 |
|  | METTL7A | 0.591712634 | 1.79E-35 | RALY | -0.520951152 | 1.67E-26 |
|  | AGXT2 | 0.591102902 | 2.19E-35 | ORC6L | -0.520076261 | 2.09E-26 |
|  | CYB5A | 0.590317106 | 2.83E-35 | KIF18B | -0.519749919 | 2.27E-26 |
|  | SEPP1 | 0.589282113 | 3.96E-35 | CKAP4 | -0.518835246 | 2.88E-26 |
|  | CYP8B1 | 0.589071515 | 4.25E-35 | CCNF | -0.518477656 | 3.15E-26 |
|  | SLC22A1 | 0.585687399 | 1.27E-34 | HMGA1 | -0.518078954 | 3.49E-26 |
|  | SEC14L2 | 0.585457728 | 1.37E-34 | NAP1L1 | -0.517931471 | 3.63E-26 |
|  | CYP4F3 | 0.58497212 | 1.60E-34 | NUF2 | -0.517554265 | 3.99E-26 |
|  | AGXT2L1 | 0.584306266 | 1.98E-34 | SGOL2 | -0.517536041 | 4.01E-26 |
|  | CFI | 0.58357755 | 2.50E-34 | KIF15 | -0.517238233 | 4.33E-26 |
|  | C1R | 0.58272338 | 3.28E-34 | FAM54A | -0.517055579 | 4.54E-26 |
|  | C4orf34 | 0.581661746 | 4.59E-34 | GINS1 | -0.516722283 | 4.94E-26 |
|  | RNASE4 | 0.581630289 | 4.64E-34 | HMGA2 | -0.516600798 | 5.09E-26 |
|  | CES2 | 0.579622481 | 8.75E-34 | C2orf29 | -0.515857251 | 6.15E-26 |
|  | PCK2 | 0.57924231 | 9.87E-34 | TRIM59 | -0.514084855 | 9.65E-26 |
|  | ALDH2 | 0.578670917 | 1.18E-33 | KIF18A | -0.513298507 | 1.18E-25 |
| C7 | LAMA2 | 0.877217356 | 1.89E-116 | SLC26A6 | -0.463110394 | 1.37E-20 |
|  | DCN | 0.874778159 | 5.13E-115 | EME1 | -0.44273638 | 9.23E-19 |
|  | MFAP4 | 0.834474027 | 6.15E-95 | ESM1 | -0.439246553 | 1.85E-18 |
|  | SVEP1 | 0.827131327 | 7.31E-92 | C9orf100 | -0.436462466 | 3.19E-18 |
|  | GGT5 | 0.808407786 | 1.22E-84 | FBXO43 | -0.431322275 | 8.66E-18 |
|  | PODN | 0.806316105 | 6.99E-84 | STRA13 | -0.43124956 | 8.78E-18 |
|  | OMD | 0.804791282 | 2.46E-83 | PTTG1 | -0.426738107 | 2.08E-17 |
|  | LUM | 0.803554577 | 6.77E-83 | TK1 | -0.425452696 | 2.65E-17 |
|  | FBLN2 | 0.802887743 | 1.16E-82 | TRAIP | -0.424345899 | 3.27E-17 |
|  | NGFR | 0.800984829 | 5.42E-82 | NCAPG | -0.418031561 | 1.06E-16 |
|  | LAMC3 | 0.798908084 | 2.85E-81 | UBE2T | -0.417622703 | 1.14E-16 |
|  | OGN | 0.798152721 | 5.18E-81 | SKA1 | -0.414908055 | 1.87E-16 |
|  | DPT | 0.798097157 | 5.41E-81 | CHEK1 | -0.414340872 | 2.08E-16 |
|  | PRELP | 0.795387113 | 4.54E-80 | CCDC137 | -0.412618751 | 2.84E-16 |
|  | EFEMP1 | 0.795110825 | 5.62E-80 | BIRC5 | -0.412279603 | 3.02E-16 |
|  | SHISA3 | 0.790954597 | 1.37E-78 | CDC25C | -0.40807292 | 6.43E-16 |
|  | INMT | 0.790046204 | 2.73E-78 | EIF2B4 | -0.40742028 | 7.22E-16 |
|  | COL14A1 | 0.788854991 | 6.70E-78 | CDCA3 | -0.406539592 | 8.44E-16 |
|  | AEBP1 | 0.786328137 | 4.41E-77 | CDC20 | -0.405904389 | 9.44E-16 |
|  | TCF21 | 0.782753755 | 6.07E-76 | SUV39H1 | -0.404669975 | 1.17E-15 |
|  | ASPN | 0.782213638 | 8.99E-76 | CCNB1 | -0.40202576 | 1.87E-15 |
|  | THBS2 | 0.778717196 | 1.11E-74 | KIF2C | -0.401829126 | 1.93E-15 |
|  | CRISPLD2 | 0.777354708 | 2.91E-74 | HOXD10 | -0.401238653 | 2.14E-15 |
|  | HAND2 | 0.774965601 | 1.56E-73 | MXD3 | -0.400159053 | 2.58E-15 |
|  | EPHA3 | 0.77416096 | 2.73E-73 | AZI1 | -0.39968999 | 2.80E-15 |
|  | NBLA00301 | 0.774080706 | 2.88E-73 | POC1A | -0.395953125 | 5.33E-15 |
|  | ZFPM2 | 0.770092322 | 4.48E-72 | TROAP | -0.392170503 | 1.01E-14 |
|  | PCDH7 | 0.764795671 | 1.57E-70 | SPC25 | -0.389880616 | 1.49E-14 |
|  | EMILIN1 | 0.762983181 | 5.19E-70 | FATE1 | -0.389602963 | 1.56E-14 |
|  | TMEM132E | 0.761402177 | 1.46E-69 | KIF4A | -0.389240414 | 1.66E-14 |
|  | CCL21 | 0.760484044 | 2.65E-69 | TAF9 | -0.388150474 | 1.99E-14 |
|  | FBLN5 | 0.760196666 | 3.20E-69 | AATF | -0.387562447 | 2.19E-14 |
|  | GALNTL1 | 0.755266936 | 7.50E-68 | CENPA | -0.387293749 | 2.29E-14 |
|  | PDZRN4 | 0.751115274 | 1.01E-66 | AAAS | -0.385493376 | 3.09E-14 |
|  | GAS1 | 0.746849483 | 1.38E-65 | UCK2 | -0.385029713 | 3.34E-14 |
|  | COLEC10 | 0.746301509 | 1.93E-65 | HGS | -0.384358677 | 3.73E-14 |
|  | PLCXD3 | 0.742488942 | 1.91E-64 | CDT1 | -0.384309918 | 3.76E-14 |
|  | SPON1 | 0.741004352 | 4.60E-64 | E2F6 | -0.384214701 | 3.81E-14 |
|  | IGFBP7 | 0.741004208 | 4.60E-64 | SIRT7 | -0.382439783 | 5.10E-14 |
|  | FXYD6 | 0.735858741 | 9.31E-63 | RACGAP1 | -0.382054733 | 5.43E-14 |
|  | PTH1R | 0.734813403 | 1.70E-62 | CENPH | -0.381525115 | 5.92E-14 |
|  | TEK | 0.734657265 | 1.86E-62 | HSPB11 | -0.381092273 | 6.35E-14 |
|  | PTGIR | 0.734143263 | 2.50E-62 | HOXD9 | -0.380828599 | 6.63E-14 |
|  | BGN | 0.733407367 | 3.80E-62 | NDC80 | -0.380706367 | 6.77E-14 |
|  | BHLHE22 | 0.730478996 | 2.00E-61 | PSMC3IP | -0.380091456 | 7.48E-14 |
|  | ALDH1A3 | 0.724307513 | 6.19E-60 | NUF2 | -0.379173484 | 8.67E-14 |
|  | TNXB | 0.718671723 | 1.31E-58 | CENPM | -0.378785681 | 9.23E-14 |
|  | LRRC32 | 0.718611343 | 1.35E-58 | SLC25A19 | -0.378578414 | 9.55E-14 |
|  | C1QTNF7 | 0.717161679 | 2.93E-58 | KIFC1 | -0.378369066 | 9.87E-14 |
|  | LTBP4 | 0.715126304 | 8.61E-58 | NARF | -0.377737034 | 1.09E-13 |
| CFP | CD5L | 0.749266893 | 3.16E-66 | MYO19 | -0.397793461 | 3.89E-15 |
|  | CETP | 0.704287496 | 2.28E-55 | TMEM209 | -0.358662858 | 2.12E-12 |
|  | S1PR4 | 0.689413656 | 3.23E-52 | DARS2 | -0.351228001 | 6.41E-12 |
|  | CD72 | 0.688063378 | 6.11E-52 | SLC35E3 | -0.347333519 | 1.13E-11 |
|  | PDE6G | 0.683997548 | 4.08E-51 | CENPL | -0.346941928 | 1.20E-11 |
|  | LST1 | 0.679155912 | 3.75E-50 | C5orf51 | -0.346834308 | 1.21E-11 |
|  | FCN1 | 0.678626517 | 4.77E-50 | PRKCA | -0.344885916 | 1.61E-11 |
|  | CD48 | 0.668142332 | 5.01E-48 | XRCC2 | -0.339737129 | 3.34E-11 |
|  | GZMK | 0.666635566 | 9.62E-48 | CLCN2 | -0.336179823 | 5.49E-11 |
|  | GMFG | 0.665056949 | 1.90E-47 | SUN1 | -0.33439448 | 7.03E-11 |
|  | GIMAP1 | 0.663056724 | 4.47E-47 | WDR45L | -0.334394155 | 7.03E-11 |
|  | C1orf162 | 0.662373162 | 5.98E-47 | CKAP2L | -0.334285341 | 7.13E-11 |
|  | CST7 | 0.661461956 | 8.80E-47 | UCHL5 | -0.333561904 | 7.88E-11 |
|  | LILRB2 | 0.660094674 | 1.57E-46 | HMMR | -0.333545291 | 7.90E-11 |
|  | P2RY13 | 0.655770123 | 9.56E-46 | VEGFA | -0.333487666 | 7.96E-11 |
|  | NAPSB | 0.654799203 | 1.43E-45 | CNNM4 | -0.332032849 | 9.72E-11 |
|  | LILRA5 | 0.652351004 | 3.91E-45 | DUS4L | -0.330172112 | 1.25E-10 |
|  | HCLS1 | 0.652138006 | 4.27E-45 | OCLN | -0.329093276 | 1.45E-10 |
|  | SPIC | 0.648524874 | 1.85E-44 | ANLN | -0.329079257 | 1.45E-10 |
|  | MPEG1 | 0.648147761 | 2.15E-44 | KIF14 | -0.328998117 | 1.47E-10 |
|  | CD247 | 0.638781457 | 8.78E-43 | PRC1 | -0.32740322 | 1.82E-10 |
|  | MARCO | 0.636323515 | 2.27E-42 | GPSM2 | -0.325724292 | 2.28E-10 |
|  | CLEC10A | 0.635488711 | 3.13E-42 | C1orf9 | -0.323330436 | 3.14E-10 |
|  | MYO1F | 0.63534934 | 3.31E-42 | PALB2 | -0.323063005 | 3.25E-10 |
|  | EMR1 | 0.634256771 | 5.03E-42 | LOC387646 | -0.322835073 | 3.35E-10 |
|  | TBX21 | 0.633803978 | 5.98E-42 | GTF2IRD1 | -0.322192993 | 3.65E-10 |
|  | IL16 | 0.630956731 | 1.76E-41 | CENPF | -0.321047349 | 4.24E-10 |
|  | CCL23 | 0.630581948 | 2.03E-41 | RPRD2 | -0.320799277 | 4.38E-10 |
|  | GIMAP5 | 0.630073756 | 2.46E-41 | TFRC | -0.319756472 | 5.03E-10 |
|  | WAS | 0.629830618 | 2.69E-41 | VSIG10 | -0.319058478 | 5.51E-10 |
|  | GFRA2 | 0.629778093 | 2.75E-41 | PLS1 | -0.316918757 | 7.27E-10 |
|  | SLAMF6 | 0.628728343 | 4.08E-41 | NUP155 | -0.316525039 | 7.65E-10 |
|  | PRKCB | 0.625940566 | 1.15E-40 | PIK3CB | -0.316423608 | 7.75E-10 |
|  | ARHGAP15 | 0.624855792 | 1.72E-40 | MAST2 | -0.315114444 | 9.18E-10 |
|  | ZNF831 | 0.624778099 | 1.77E-40 | METTL2A | -0.314441841 | 1.00E-09 |
|  | ABI3 | 0.62430813 | 2.11E-40 | ZMYM1 | -0.313852116 | 1.08E-09 |
|  | LILRB1 | 0.623918685 | 2.43E-40 | RMI1 | -0.312886595 | 1.22E-09 |
|  | CLEC12A | 0.623644773 | 2.69E-40 | CENPI | -0.312687685 | 1.25E-09 |
|  | DPEP2 | 0.623597654 | 2.74E-40 | ZDHHC23 | -0.312479061 | 1.29E-09 |
|  | KLRB1 | 0.622289089 | 4.43E-40 | NEK2 | -0.312248456 | 1.32E-09 |
|  | C1QA | 0.622225042 | 4.53E-40 | MAP3K9 | -0.311901075 | 1.38E-09 |
|  | CXCR2P1 | 0.621190909 | 6.62E-40 | TSSK6 | -0.311666536 | 1.43E-09 |
|  | PLCB2 | 0.619743299 | 1.12E-39 | INADL | -0.311629745 | 1.43E-09 |
|  | GPSM3 | 0.619268226 | 1.33E-39 | KIAA1522 | -0.311348093 | 1.48E-09 |
|  | GIMAP4 | 0.618124801 | 2.01E-39 | DEPDC1 | -0.311225119 | 1.51E-09 |
|  | CD33 | 0.617180884 | 2.83E-39 | GATSL1 | -0.311096892 | 1.53E-09 |
|  | COTL1 | 0.616968625 | 3.05E-39 | NCAPG2 | -0.310605522 | 1.63E-09 |
|  | SIGLEC7 | 0.616684585 | 3.38E-39 | CCDC21 | -0.310171473 | 1.72E-09 |
|  | DOK2 | 0.616521716 | 3.58E-39 | IQCC | -0.309717161 | 1.82E-09 |
|  | SH2D1A | 0.61608701 | 4.19E-39 | IQGAP3 | -0.309039241 | 1.99E-09 |
| CFHR3 | CFHR4 | 0.765413186 | 1.04E-70 | SPATS2 | -0.490576924 | 2.93E-23 |
|  | CFHR1 | 0.734621949 | 1.90E-62 | PIGS | -0.479058825 | 4.13E-22 |
|  | CFH | 0.721068752 | 3.61E-59 | TCF3 | -0.465987564 | 7.37E-21 |
|  | CFHR2 | 0.683813331 | 4.44E-51 | FRAS1 | -0.460169928 | 2.55E-20 |
|  | HP | 0.675690339 | 1.79E-49 | CS | -0.458159141 | 3.90E-20 |
|  | HPX | 0.668511294 | 4.26E-48 | GTF3C2 | -0.457833836 | 4.18E-20 |
|  | ITIH4 | 0.647367752 | 2.95E-44 | C17orf63 | -0.457580951 | 4.41E-20 |
|  | C8B | 0.638584596 | 9.47E-43 | C11orf84 | -0.457509601 | 4.48E-20 |
|  | C3P1 | 0.63149745 | 1.44E-41 | ILF3 | -0.457426761 | 4.55E-20 |
|  | HPR | 0.627536688 | 6.37E-41 | TAF6 | -0.455441587 | 6.90E-20 |
|  | CFB | 0.626190776 | 1.05E-40 | NCAPD2 | -0.453272769 | 1.08E-19 |
|  | SERPINC1 | 0.623471606 | 2.87E-40 | NDRG3 | -0.451769553 | 1.48E-19 |
|  | C6 | 0.620302988 | 9.14E-40 | NKX3-2 | -0.451736779 | 1.49E-19 |
|  | F9 | 0.61815392 | 1.99E-39 | PLXNA1 | -0.451189829 | 1.66E-19 |
|  | FGA | 0.614795358 | 6.64E-39 | LMNB2 | -0.448105605 | 3.12E-19 |
|  | C8A | 0.614320193 | 7.86E-39 | ANKRD52 | -0.447098604 | 3.83E-19 |
|  | SERPING1 | 0.613807674 | 9.43E-39 | TMEM201 | -0.446567576 | 4.27E-19 |
|  | APOF | 0.61339826 | 1.09E-38 | MAPRE1 | -0.44006369 | 1.57E-18 |
|  | ANXA10 | 0.60874713 | 5.60E-38 | GTF2IRD1 | -0.432918225 | 6.36E-18 |
|  | C1S | 0.608182612 | 6.82E-38 | PKM2 | -0.432817754 | 6.49E-18 |
|  | HAO1 | 0.608021712 | 7.21E-38 | RALY | -0.428589628 | 1.46E-17 |
|  | FGG | 0.605732389 | 1.59E-37 | BCL9L | -0.426183855 | 2.31E-17 |
|  | HSD17B6 | 0.604339006 | 2.58E-37 | ABI2 | -0.425615563 | 2.57E-17 |
|  | MASP2 | 0.596000433 | 4.33E-36 | SMARCD1 | -0.424012153 | 3.48E-17 |
|  | TFR2 | 0.594365756 | 7.46E-36 | SMPD4 | -0.423967087 | 3.51E-17 |
|  | FGB | 0.594316023 | 7.58E-36 | FBXL19 | -0.423484773 | 3.84E-17 |
|  | MAT1A | 0.593338528 | 1.05E-35 | E2F6 | -0.422373744 | 4.73E-17 |
|  | DHODH | 0.590357959 | 2.79E-35 | MTA3 | -0.421371702 | 5.70E-17 |
|  | ACSM2A | 0.588997829 | 4.35E-35 | CBX1 | -0.418566471 | 9.59E-17 |
|  | GYS2 | 0.587401929 | 7.30E-35 | AACS | -0.418054016 | 1.05E-16 |
|  | CFHR5 | 0.585028647 | 1.57E-34 | MFSD10 | -0.416874707 | 1.31E-16 |
|  | ITIH1 | 0.584773345 | 1.70E-34 | KIAA1841 | -0.416547808 | 1.39E-16 |
|  | SERPINA10 | 0.584070426 | 2.13E-34 | STRN4 | -0.415666502 | 1.63E-16 |
|  | AGXT2 | 0.582686465 | 3.32E-34 | CCDC97 | -0.414187457 | 2.14E-16 |
|  | ACSM2B | 0.582082205 | 4.02E-34 | RAVER1 | -0.4139426 | 2.23E-16 |
|  | GLYATL1 | 0.580946141 | 5.76E-34 | COPS7B | -0.412780165 | 2.76E-16 |
|  | RBP4 | 0.577727542 | 1.59E-33 | TYRO3 | -0.412494145 | 2.91E-16 |
|  | F13B | 0.575981229 | 2.74E-33 | PPP1R14C | -0.41204785 | 3.15E-16 |
|  | SAA4 | 0.569503488 | 2.01E-32 | YEATS2 | -0.412019939 | 3.17E-16 |
|  | HFE2 | 0.565474902 | 6.79E-32 | UBAP2 | -0.41097727 | 3.82E-16 |
|  | A1BG | 0.565216614 | 7.34E-32 | C3orf21 | -0.410476346 | 4.18E-16 |
|  | ANG | 0.564439816 | 9.26E-32 | LARP4B | -0.409135316 | 5.32E-16 |
|  | HRSP12 | 0.564302011 | 9.65E-32 | RACGAP1 | -0.408912627 | 5.53E-16 |
|  | BDH1 | 0.564080536 | 1.03E-31 | LIMK1 | -0.407968434 | 6.55E-16 |
|  | ITIH3 | 0.562092784 | 1.86E-31 | TPD52L2 | -0.407857852 | 6.68E-16 |
|  | SLC10A1 | 0.558516007 | 5.35E-31 | DVL3 | -0.407501645 | 7.11E-16 |
|  | FETUB | 0.55835108 | 5.61E-31 | UBA2 | -0.406885463 | 7.94E-16 |
|  | RDH16 | 0.557402184 | 7.41E-31 | BEND3 | -0.406447984 | 8.58E-16 |
|  | LRG1 | 0.555059676 | 1.47E-30 | C9orf140 | -0.406442461 | 8.58E-16 |
|  | CYP4A11 | 0.553421312 | 2.35E-30 | PRR12 | -0.406325774 | 8.76E-16 |
